# Impacts of *Salmonella enterica* Serovar Typhimurium and Its *speG* Gene on the Transcriptomes of *In Vitro* M Cells and Caco-2 Cells

**DOI:** 10.1371/journal.pone.0153444

**Published:** 2016-04-11

**Authors:** Ke-Chuan Wang, Chih-Hung Huang, Ching-Jou Huang, Shiuh-Bin Fang

**Affiliations:** 1 Division of Pediatric Gastroenterology and Hepatology, Department of Pediatrics, Shuang Ho Hospital, Taipei Medical University, Taipei, Taiwan; 2 Department of Pediatrics, School of Medicine, College of Medicine, Taipei Medical University, Taipei, Taiwan; 3 Graduate Institute of Biochemical and Biomedical Engineering, National Taipei University of Technology, Taipei, Taiwan; University of Hong Kong, HONG KONG

## Abstract

Microfold or membranous (M) cells are specialized intestinal epithelial cells responsible for host immunity. The *speG* mutant of *Salmonella* Typhimurium (*S*. Typhimurium) is a nonreplicating strain within human cells to be a candidate vaccine vector for interacting with M cells. We conducted this study to identify the genes are differently expressed between *in vitro* M cells and Caco-2 cells, and to determine whether *S*. Typhimurium and *speG* affect the transcriptomes of both cell types. *In vitro* M cells and Caco-2 cells were infected with wild-type (WT) *S*. Typhimurium, its Δ*speG* mutant, or none for 1 h for RNA microarrays; the transcriptomes among the 6 pools were pairwisely compared. Genetic loci encoding scaffold (e.g., *HSCHR7_CTG4_4*, *HSCHR9_CTG9_35*), long noncoding RNA, membrane-associated protein (*PITPNB*), neuron-related proteins (*OR8D1*, *OR10G9*, and *NTNG2*), and transporter proteins (*MICU2* and *SLC28A1*) were significantly upregulated in uninfected M cells compared with uninfected Caco-2 cells; and their encoding proteins are promising M-cell markers. Significantly upregulated *HSCHR7_CTG4_4* of uninfected *in vitro* M cells were *speG*-independently downregulated by *S*. Typhimurium infection that is a remarkable change representing an important but unreported characteristic of M cells. The immune responses of *in vitro* M cells and Caco-2 cells can differ and reply on *speG* or not, with *speG*-dependent regulation of *KYL4*, *SCTR*, *IL6*, *TNF*, and *CELF4* in Caco-2 cells, *JUN*, *KLF6*, and *KCTD11* in M cells, or *speG*-independent modulation of *ZFP36* in both cells. This study facilitates understanding of the immune responses of *in vitro* M cells after administering the *S*. Typhimurium Δ*speG* mutant as a future vaccine vector.

## Introduction

Microfold or membranous (M) cells, are specialized intestinal epithelial cells that are involved in gut immunity that relies on collaboration between antigen-sampling of M cells and lymphoid or dendritic cells; therefore, M cells could a good target for delivery of mucosal vaccines into hosts for inducing cellular and humoral immunity [1]. M cells reside in 10% of epithelial cells within the follicle-associated epithelium (FAE) overlaid on the lymphoid follicles of gut-associated lymphoid tissue, including Peyer’s patches, and isolated lymphoid follicles or solitary intestinal lymphoid tissue as non-FAE intestinal villous M cells [2]. M cells are crucial for gut immunity because pathogens and macromolecules within the intestinal lumen can transcytose across M cells into the submucosa of Peyer’s patches to interact with antigen presenting cells and activate subsequent immune responses [2]. The intestinal epithelium consists of 6 major cell types: absorptive columnar epithelial cells, mucin-secreting goblet cells, enteroendocrine cells, antimicrobial peptide-secreting Paneth cells, undifferentiated cells, and M cells [3]. The intestinal epithelial cells constitute a host defense barrier against pathogens during enteric infection. Tight junctions among intestinal epithelial cells, the unique organelles localized to the apical-lateral region of the intestinal epithelium, can block the movement of macromolecules and pathogens across the intestinal epithelium to its basolateral side [4]. However, M cells have sparse irregular microvilli apically, and pocket-like cytoplasmic invagination harboring immune cells basolaterally. These distinctive morphological features enable M cells to uptake and transcytose intestinal antigens to underlying lymphoid tissues where antigen-presenting cells can present the internalized antigens to T cells for initiating protective immune responses [2].

Information on the mechanisms of M cell differentiation remains scant. A few studies have indicated that the epithelial–mesenchymal transition (EMT)-regulating transcription factor Slug, receptor activator of nuclear factor-κB (NF-κB) ligand (RANKL), and SpiB might mediate M-cell development [5, 6]. Primary epithelial cells cultured from FAE isolated from bovine terminal rectum and intestinal epithelial cells in the murine ligated ileal loops containing Peyer’s pactches can be transformed into M cells by *S*. Typhimurium that SopB-dependently activates Wnt/β-catenin signaling leading to induction of both RANKL and its receptor RANK [6]. The expression of the M cell-specific marker vimentin expression can be controlled via the Wnt/β-catenin signaling pathway following an association with Slug [7]. In the mice studies, M cells located over Peyer’s patches might be derived from Lgr5 stem-cell-derived epithelial cells, and the development of M cells requires the RANKL-induced expression of SpiB [8] or the SpiB-independent pathway after *S*. Typhimurium infection [9]. However, the detailed mechanism underlying M-cell development requires further clarification.

Because human M cells are typically unapproachable, and *in vivo* M cells in animal studies cannot be used to answer all questions regarding human M cells, a convenient *in vitro* human M-cell model is required. Therefore *in vitro* M cells were firstly established by coculturing the human colon carcinoma cells line, Caco-2, with lymphocytes isolated from Peyer’s patches of BALB/c mice, and the increased internalization of bacteria was demonstrated in this model [10]. Thereafter, a modified *in vitro* M-cell model was established by coculturing Caco-2 cells with human Raji B cells [11], thus providing a simple method for investigating human M cells. However, little is known regarding the mechanisms underlying this *in vitro* model.

*Salmonella* is one of the enteropathogenic bacteria that can penetrate the intestinal epithelial barrier through M cells from the intestinal lumen into the lamina propria. However, whether *Salmonella* preferentially invades M cells rather than enterocytes in humans remains obscure because so far no human study in this issue has been conducted. Animal studies have been used to demonstrate that *Salmonella enterica* serovar Typhimurium (*S*. Typhimurium) preferably invades M cells located in the FAE of Peyer’s patches in mice [12, 13]. These M cells are a critical entry site for the colonization and internalization of *Salmonella* in *Salmonella* pathogenesis. However, the cellular responses of M cells after *Salmonella* infection remain unclear.

To date, the phenotypic characterization of *speG* has not been investigated in *Salmonella* within human intestinal epithelial cells although the expression of *speG* has been annotated in the previous studies using RNA-sequencing. The expression of *speG* in *S*. Typhimurium was downregulated in some of the 22 *in vitro* infection-relevant environmental conditions, particularly late stationary phase and pH3 shock [14]. The RNA transcriptomic expression of *speG* was non-significantly expressed at early stationary phase of *S*. Typhimurium [15]. However, the small regulatory RNAs of *speG* were non-significantly expressed in *S*. Typhimurium after invasion into murine macrophages for 18 h [16]. In all *Shigella* species, *sepG* encodes spermidine N1-acetyltransferase mediating intracellular spermidine accumulation [17]. Another study using human non-intestinal epithelial cells, *Caenorhabditis elegans*, and C57/BL6 mice (Nramp-) demonstrated that polyamines are required for virulence in *S*. Typhimurium, which is especially controlled by putrescine and spermidine through the stimulation of expression of their biosynthetic genes at 5 distinct genetic loci: *speA*, *speB*, *speC*, *speDE*, and *speF*, and these polyamines function as a signal priming *S*. Typhimurium for intracellular survival [18]. Our preliminary results revealed that deletion of *speG* can attenuate intracellular replication but not colonization and invasion of *S*. Typhimurium in human epithelial cells [19]. Therefore, this characteristic enables the *S*. Typhimurium *speG* mutant to function as a potential oral vaccine vector.

For this study, we used the well established *in vitro* M-cell model as previously described [20]. The *in vitro* M cells and Caco-2 cells were infected with wild-type (WT) *S*. Typhimurium and *speG*-deleted strains. The transcriptomes of *in vitro* M cells and Caco-2 cells before and after *S*. Typhimurium infection were obtained using RNA microarrays, after which we conducted a pairwise comparison. We identified significantly regulated genes of the *in vitro* M cells compared with Caco-2 cells, those of both cells after *S*. Typhimurium infection relative to non-infected cells, and those of both the cells after the depletion of *speG* compared with the WT strain. These results were then confirmed by conducting a quantitative real-time polymerase chain reaction (qRT-PCR) of the selected identified genes. Overall, this microarray study enables a thorough investigation of the genes involved in the differentiation of *in vitro* M cells, and furthers our understanding regarding the gene expression of *in vitro* M cells and Caco-2 cells that can be postinfectiously regulated by *S*. Typhimurium and its *speG* gene.

## Materials and Methods

### Bacterial strains and culture

The *S*. Typhimurium WT strain SL1344, *Salmonella* pathogenicity island-1 (SPI-1) mutant strain Δ*spaS*, and *speG*-deleted strain Δ*speG* were used in this study. The SPI-1 mutant strain Δ*spaS* was provided by Professor Duncan Maskell. The *speG* gene in *S*. Typhimurium SL1344 was deleted using the lambda Red recombinase-mediated integration of linear PCR amplicons as previously described [21], with the gene replaced by a kanamycin-resistance gene. Before each experiments, these strains were recovered from GermBank (CMP^TM^ Culture Media, Taiwan) stocks maintained at −80°C onto Luria–Bertani (LB) agar plates, which were subsequently incubated at 37°C for growing colonies. For cell infections, the single colonies of each strains were aerobically grown in 3 mL of Luria–Bertani (LB) broth (Difco) at 37°C for 18 h to reach a bacterial density of 1 × 10^9^ CFU/mL as overnight cultures. If necessary, kanamycin (50 μg/mL) was used for maintaining Δ*spaS* and Δ*speG*.

### *In vitro* M-cell culture model

The *in vitro* M-cell culture model was established as previously described, with minor modifications [20]. The Raji B cells, a cell line of human Burkitt’s lymphoma purchased from the Bioresource Collection and Research Center, Taiwan (BCRC No. 60116, originally from ATCC No. CCL-86), were grown in 90% RPMI 1640 medium (Gibco) supplemented with 10% fetal bovine serum (FBS, Gibco), and 1% L-glutamine (Gibco), 1.5 g/L of sodium bicarbonate (Sigma), 4.5 g/L of glucose (Sigma), 10 mM of HEPES (Sigma), and 1.0 mM of sodium pyruvate (Sigma) at 37°C in 5% CO_2_. The Caco-2 cells, a C2BBe1 clone of a Caucasian human’s colon adenocarcinoma, purchased from the Bioresource Collection and Research Center, Taiwan (BCRC No. 60182, originally from ATCC No. CRL-2102), were grown in Dulbecco’s modified Eagle’s medium (DMEM; Gibco) supplemented with 10% FBS (Sigma), 100 μM of nonessential amino acids (Sigma), 1% L-glutamine (Gibco), and 0.01 mg/mL of transferrin (Sigma) at 37°C in 5% CO_2_. After trypsinization, 5 × 10^5^ Caco-2 cells (3–6 passages since obtainment from BCRC) were seeded onto the 9.5-mm-diameter collagen (10 μg/cm^2^)-precoated polycarbonate Millipore membrane inserts with 3-μm pores in Millicell 24-well plates, and were incubated at 37°C in 5% CO_2_ for 14 d. At this stage, the transepithelial electrical resistance (TEER) values across the Caco-2 cell monolayers were measured using a voltage meter EVOM (World Precision Instruments) in accordance with manufacturer instructions. After the polarization of Caco-2 cells with adequate epithelial barrier integrity was achieved and validated using TEER values higher than 300 Ω·cm^2^, Raji B cells at a density of 5 × 10^5^ cells/well were added into the basolateral chamber, which contained one-third of the complete RPMI medium and two-thirds of the complete DMEM. The co-cultures were continued for an additional 6 d to induce M-cell-like monolayer formation. During the 20-d cultures, 100 units/mL of penicillin and 0.1 mg/mL of streptomycin (Sigma) were administered in the culture media and removed 2 h before the experiments. The 20-d-old polarized Caco-2 cells that were not cocultured with Raji B cells were used as the controls.

### Electron micrographs of *in vitro* M cells and Caco-2 cells

*In vitro* M cells and Caco-2 cells were morphologically evaluated using electron microscopy after 20 d of culturing. During harvesting, each well of the cell monolayers was washed with PBS twice and fixed with 2.5% glutaraldehyde in a 0.1-M phosphate buffer for further processing before analysis through scanning electron microscopy (SEM) and transmission electron microscopy (TEM).

For SEM, the glutaraldehyde-fixed samples were washed with a 0.1-M phosphate buffer supplemented with 3% sucrose (Sigma) for 60 min. The samples were subsequently treated with 1% aqueous osmium tetroxide (Sigma) for 15 min. After treatment, the samples were dehydrated in acidified 2,2-dimethoxypropoane (Sigma) for 5 min, and transferred to 100% ethanol (Sigma) for 2 min. All of the samples were then critically point-dried in liquid CO_2_ by using a K850 critical point dryer (Quorum Technologies), mounted on aluminum stubs, and putter-coated with a mixture of gold and palladium by using an Eiko IB-2 ion coater (Eiko). Finally, these prepared samples were observed using a Hitachi SU3500 scanning electron microscope.

For TEM, the glutaraldehyde-fixed samples were post-fixed in 1% osmium tetroxide for 2 h, washed with PBS, dehydrated in a graded series of ethanol, and finally rinsed with propylene oxide (Sigma). The samples were then embedded in Epon (Serva), and incubated at 65°C for 48 h. The thick sections were cut using an ultra-microtome (0.05 μm) and mounted on mesh grids. Finally, the sections were observed under the Hitachi H-600 or HT-7700 transmission electron microscope.

### Transcytosis assay

The *in vitro* M cells were functionally assessed using a transcytosis assay. In brief, the overnight cultures of WT *S*. Typhimurium and Δ*spaS* were diluted 1:100 in fresh LB broth and incubated with shaking for 3.5 h at 37°C until the mid-log phase of growth was reached. After replacement with the serum-free media, the mid-log cultures of the bacterial strains were coincubated with *in vitro* M cells or Caco-2 cells at a multiplicity of infection (MOI) of 25 for 1 h in three independent experiments. After 1-h bacterial infection, the integrity of the infected cell monolayers were validated by their TEER values of >250 Ω·cm^2^. The mid-log cultures and media from the basolateral chambers were then serially diluted and plated out on LB agar plates prior to overnight incubation at 37°C. The bacterial colony-forming units (CFUs) were counted, and the number of the transcytosed bacteria was calculated compared with the initial inoculums, expressed as CFU per initial inoculum of 10^8^ CFU.

### Treatment of *in vitro* M cells and Caco-2 cells for RNA isolation

Using the same protocol as that used for the transcytosis assay, the *in vitro* M cells and Caco-2 cells were treated with WT *S*. Typhimurium and Δ*speG* at an MOI of 25, or with the LB broth as the noninfection control, for 1 h in two independent experiments. The total RNAs of both the cells from these 3 conditions were isolated using TRIzol reagent (Invitrogen) in accordance with the manufacturer instructions, and the preformed RNA was purified using an RNeasy Mini Kit (Qiagen). The RNA concentration was determined using a NanoDrop ND-1000 spectrophotometer (Thermo Scientific), and the integrity of the RNA samples was validated using the ratio of absorbance at 260 nm and 280 nm, as well as the RNA integrity number determined using Bioanalyzer 2100 (Agilent Technology) with an RNA 6000 Nano LabChip kit (Agilent).

### RNA microarrays

The total RNA samples were reverse-transcribed to cDNAs and subsequent cRNA, which were subsequently labeled with Cy3-CTP (CyDye, Agilent) by using an Agilent Low Input Quick-Amp Labeling kit (Agilent) in accordance with manufacturer instructions. Thus, the Cy3-labled cRNAs were fragmented into 50–100 nucleotides through incubation with a fragmentation buffer at 60°C for 30 min. All of the fragmented labeled cRNA were pooled and hybridized to Agilent SurePrint G3 human V2 GE 8 × 60K arrays that had been tiled with 50 599 human gene probes (Agilent) at 65°C for 17 h. The array chips were washed, dried, and then scanned using an Agilent microarray scanner at 535 nm for Cy3-CTP. The scanned images were quantified and analyzed using Feature Extraction 10.5.1.1 software (Agilent). The background values were corrected using the spatial detrend surface value, and were normalized by quantile. Finally, the gene expression levels in each array group were analyzed using the DAVID database (http://david.ncifcrf.gov). The relative gene expression levels were compared to the mean of each array and their fold-change values were calculated. The heap map with genes in each group that upregulated or downregulated more than 2-fold was constructed based on their normalized values by using GeneSpring multiomic analysis software (Agilent). The microarray data has been deposited in GEO (http://www.ncbi.nlm.nih.gov/geo/) and is accessible via GEO Accession Number GSE73880.

### Quantitative real-time polymerase chain reaction (qRT-PCR)

The primers of the selected genes identified in the RNA microarrays were designed using Primer3 and BLAST online (http://www.ncbi.nlm.nih.gov/tools/primer-blast/), and are listed in S1 Table. The total RNAs from the *in vitro* M cells and Caco-2 cells were isolated using the TRIzol reagent (Invitrogen) in accordance with manufacturer instructions. Each microgram of isolated total RNA was treated with one units of DNase I (New England Biolabs) at 37°C for 10 min in order to remove any residual genomic DNA, and the mixtures were heat-inactivated at 75°C for 10 min. The purified RNA samples were then reverse-transcribed into cDNAs by using an iScript cDNA synthesis kit (Bio-Rad). The mixtures were incubated at 25°C for 5 min and at 42°C for 30 min before heating at 85°C for 5 min. Finally, qRT-PCR was conducted in triplicate using 50 ng of cDNA and specific primers in each reaction by using an iQ SyBr green supermix kit (Bio-Rad) on a CFX96 real time PCR system (Rio-Rad). Each reaction involved incubation at 95°C for 3 min for denaturation, followed by 40 cycles of DNA amplifications were amplified at 95°C for 15 s, at 55°C for 30 s, and at 72°C for 30 s. Three housekeeping genes, *GAPDH*, *RPLP*, and *HPRT*, were used as internal controls. The expression level of each gene was calculated using the ^ΔΔ^Ct method. To ensure *speG* can be expressed after invasion of *S*. Typhimurium into host cells, we infected Caco-2 cells with *S*. Typhimurium SL1344 at a MOI of 25 for 6 h and extracted the RNAs from the mid-log cultures and the intracellular bacteria after lysing the infected Caco-2 cells using 10% Triton X-100. Then, the RNAs were processed as above and qRT-PCR was performed using the above protocol and the designed primers (S1 Table) for quantifying the expression levels of *speG* in *S*. Typhimurium SL1344 isolated from mid-log cultures and bacterial infected Caco-2 cells, both of which were normalized against mRNA levels of 16s as internal controls. All the values of mRNA expression levels were compared to the mean of those in mid-log cultured *S*. Typhimurium SL1344 and the data were expressed as mean ± standard deviation.

### Statistical analysis

A Student’s *t*-test was performed for a between-group comparison involving the transcytosis assay and qRT-PCR in this study. A *p* value < 0.05 was considered as significant difference. Transcriptomes among the 6 pools (uninfected, SL1344-infected or Δ*speG* mutant-infected *in vitro* M cells and Caco-2 cells) were subjected to a pairwise comparison after correction through two-way ANOVA to determinate the *p* values. A *p* value < 0.01 with > 2- or < –2-fold change was considered statistically significant.

## Results

### Electron microscopy shows morphological transformation of Caco-2 cells into *in vitro* M cells

**Fig 1:**
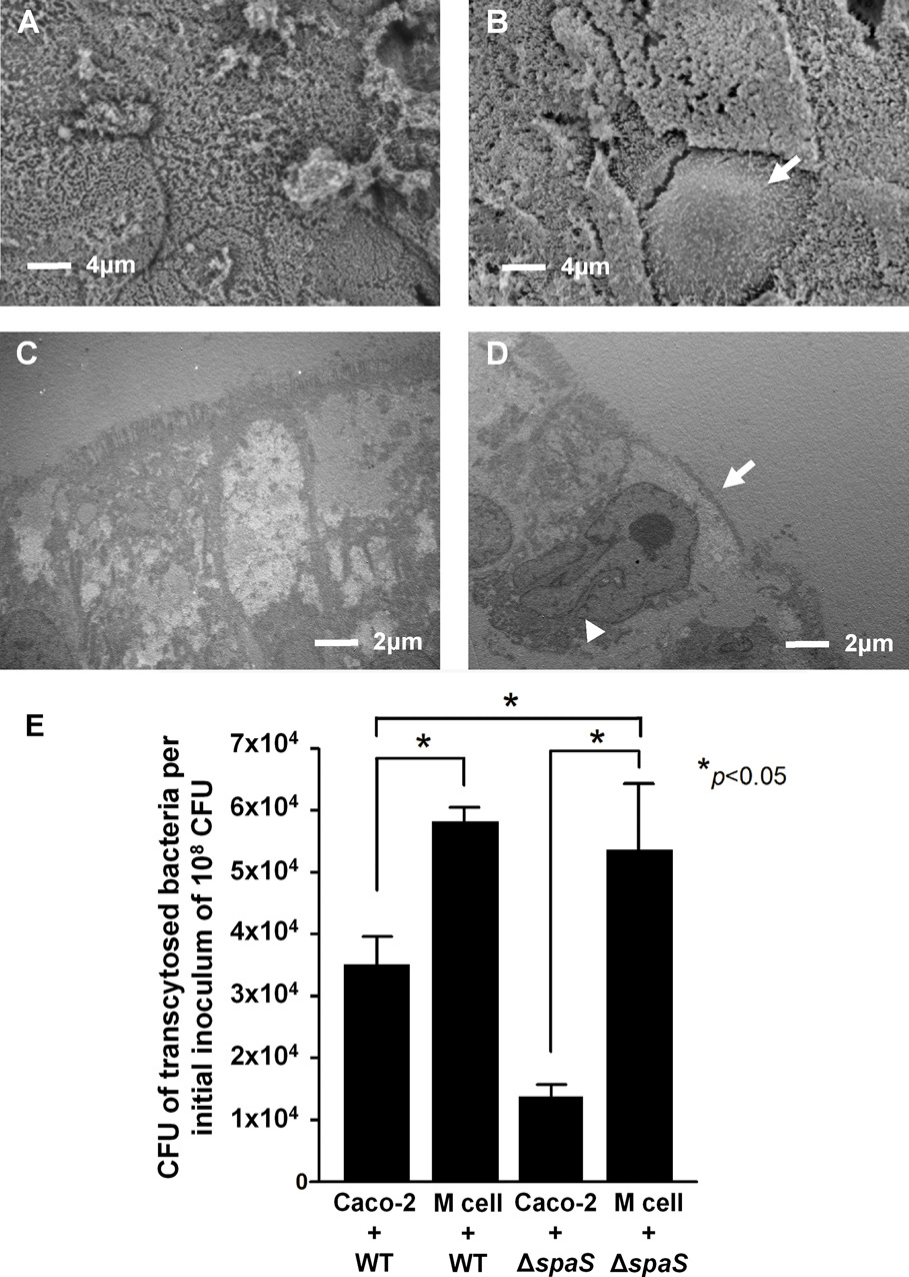
Morphological and functional assessment of polarized Caco-2 cells and *in vitro* M cells. Scanning electron micrographs (SEM) (2700×) of (A) 20-day-old polarized Caco-2 cells and (B) *in vitro* M cells. (C) Ultrastructural features of the polarized Caco-2 cell monolayer in the transmission electron micrographs (TEM). (D) Characteristic features of *in vitro* M cells in the TEM, showing irregular shortened microvilli on the apical surface (arrow) and a basolateral pocket harboring lymphocytes (arrow head). (E) Bacterial translocation rates after wild-type (WT) *S*. Typhimurium and Δ*spaS* infections in Caco-2 cell and *in vitro* M cells. *In vitro* M cells and Caco-2 cells were individually infected with WT *S*. Typhimurium and Δ*spaS* (multiplicity of infection = 25) for 1 h. The translocation rates of each group were calculated using the bacterial numbers in the media from the basolateral chambers of Transwells compared with the initial inoculums of 10^8^ colony-forming unit (CFU). *p < 0.05 was considered as statistically significant.

For confirming whether the 20-d-old polarized Caco-2 cells had been transformed into *in vitro* M cells after coculturing with Raji-B cells 6 d prior, we performed SEM and TEM to assess the morphological changes between *in vitro* M cells and Caco-2 cells. The SEM images showed that intact normal microvilli were expressed on the apical sides of the polarized Caco-2 cells (Fig 1A), and the number of irregular microvilli diminished on the cell surfaces of the *in vitro* M cells (Fig 1B). The cross-sectional TEM images showed that the lengths of microvilli and cell shape were normal in the Caco-2 cells (Fig 1C); however, the *in vitro* M cells had sparse shortened irregular microvilli on the apical surface, with basolateral invaginations harboring lymphocytes (Fig 1D). Under electron microscopy, these M-cell-like cells were not found in the Caco-2 cells as the controls, but were identified in approximately 5–10% of the polarized Caco-2 cells cocultured with Raji B cells, which is close to the previous report [2]. Therefore, these morphological characteristics confirmed that the Caco-2 cell monolayers cocultured with Raji B cells transformed into a cell population of an epithelium containing M-cell-like cells.

### Transcytosis assay proves functional translocation of noninvasive *S*. Typhimurium across *in vitro* M cells

In addition to the morphological observation of the *in vitro* M cells, we conducted a transcytosis assay to assess a critical function of M cells (i.e., to translocate particles from their apical to basolateral sides). The SPI-1 genes, such as *spaS*, of *S*. Typhimurium encode the apparatus of the type III secretion system (T3SS) which mediates bacterial colonization in the gastrointestinal tract [13, 22]. In our preliminary study, we found that *S*. Typhimurium Δ*spaS* is a noninvasive mutant strain compared with WT *S*. Typhimurium in human epithelial cells [19]. Therefore, we used this mutant strain as a noninvasive bioparticle in the transcytosis assay to determine whether it could be translocated by *in vitro* M cells. First, we observed that the transcytosis rate of *S*. Typhimurium Δ*spaS* was significantly lower than that of WT *S*. Typhimurium in the Caco-2 cells (gray versus white bar in Fig 1E). This result is consistent with our previous finding on noninvasiveness of *S*. Typhimurium Δ*spaS* [19]. In addition, our data revealed that the transcytosis rate of *S*. Typhimurium Δ*spaS* in M cells was significantly higher than that in Caco-2 cells (black versus gray bar in Fig 1E), and similar to that of WT *S*. Typhimurium in M cells (striated bar in Fig 1E). These data indicated that *in vitro* M cells can translocate this attenuated noninvasive bacteria from the apical to basolateral side, which is a major characteristic of M cells. In a similar manner, WT *S*. Typhimurium can be translocated across M cells more easily than across Caco-2 cells (striated versus white bar in Fig 1E), which could be a result of M-cell transcytosis as well as possibly a phenomenon of cell tropism for *S*. Typhimurium. Therefore, our *in vitro* M-cell culture model showed that both invasive and noninvasive *S*. Typhimurium strains could translocate across *in vitro* M-cell monolayers, which were successfully formed by transforming polarized Caco-2 cells into M-like cells with a capacity of particle transcytosis.

### Microarray analysis identifies 20 significantly upregulated genes of *in vitro* M cells compared with Caco-2 cells

**Fig 2:**
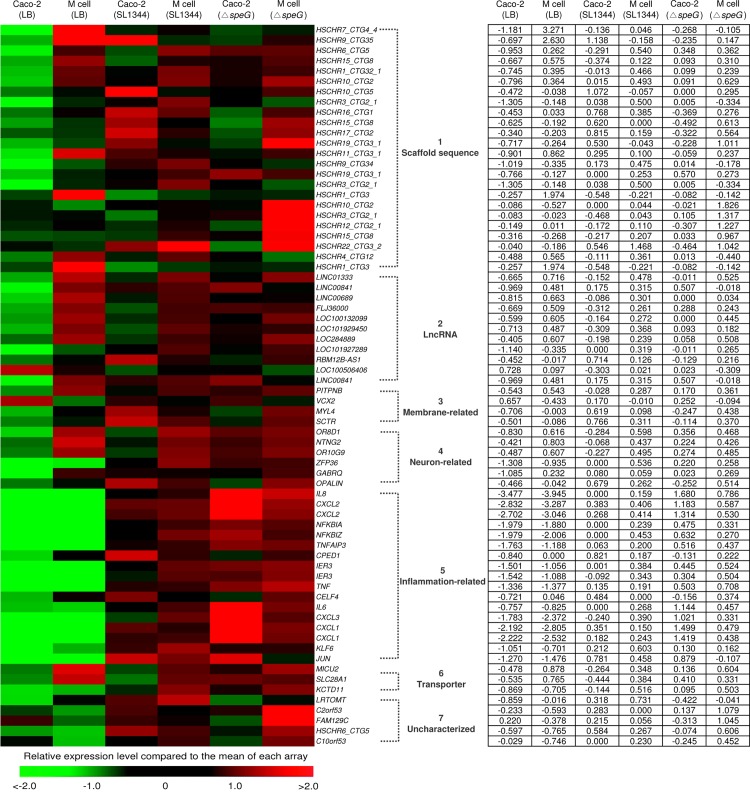
Heat map of microarray analysis and the corresponding values of fold change for the 70 significantly upregulated or downregulated genes of *in vitro* M cells and Caco-2 cells after infection with wild-type (WT) *S*. Typhimurium and Δ*speG* or remaining uninfected for 1 h. We found 70 genes of *in vitro* M cells and Caco-2 cells that were significantly upregulated (>2-fold change, red bricks) after treatment with WT *S*. Typhimurium SL1344, the *speG*-deleted mutant (Δ*speG*), and only Luria-Bertani broth (LB). These 70 genes were classified into 8 groups according to their relative or potential functions. Red bricks represent higher gene expression levels compared with average levels (black bricks).

Prior to micoarray analysis, we first confirmed that the expression level of *speG* in *S*. Typhimurium within the infected Caco-2 cells was 1.6-fold higher than in the extracellular *S*. Typhimurium from mid-log culture before invasion (S1 Fig). After we morphologically and functionally confirmed our *in vitro* M-cell culture model, we further compared the global transcriptomes between *in vitro* M cells and Caco-2 cells, and investigated the influence of *S*. Typhimurium and its *speG* gene in the host cells by using RNA microarrays. Among 50,599 genes, we identified 70 genes that were significantly upregulated or downregulated, and they were classified into 8 groups based on their relative or potential functions (Fig 2). Microarray analysis revealed that 20 genes of the uninfected *in vitro* M cells were significantly upregulated compared with the uninfected polarized Caco-2 cells, and the functions of these 20 genes were grouped into 6 different categories: 6 scaffold sequences, 7 long noncoding RNA (lncRNA) genes, one membrane-associated gene, 3 neuron-related protein-encoding genes, 2 transporter-related genes, and one uncharacterized X-linked member gene (Table 1). Among the 6 scaffold sequences belonging to the uncharacterized portion of the bacterial genome, the most significantly upregulated gene of the *in vitro* M cells was *HSCHR7_CTG4_4* (17.637-fold change, Table 1), which encodes a hypothetical protein and is located between *HTR5A* antisense RNA 1 and *PAXIP1* antisense RNA 1 on chromosome 7. Another 2 markedly upregulated scaffold sequences with limited information are *HSCHR9_CTG35* which is close to *PTPN3* (protein tyrosine phosphatase nonreceptor type 3) on chromosome 9, and *HSCHR10_CTG2*, which is located between *PITRM1* (pitrilysin metallopeptidase 1) and *LOC101927880* (lncRNA) on chromosome 10. The other 3 scaffold sequences, *HSCHR6_CTG5*, *HSCHR15_CTG8*, and *HSCHR1_CTG32_1*, encode hypothetical products with unknown functions. In addition, 7 lncRNA genetic loci were significantly upregulated in the *in vitro* M cells, with a change in RNA expression levels ranging from 2.038- to 2.622-fold and with unknown functions (Table 1). The highly expressed *PITPNB* gene (2.086-fold change) of the *in vitro* M cells encodes phosphatidylinositol transfer protein β, which catalyzes the transfer of phosphatidylinositol and phosphatidylcholine, and is a cytoplasmic protein associated with the cell membrane. Compared with the Caco-2 cells, the *in vitro* M cells had 3 significantly upregulated genes, *OR8D1*, *OR10G9*, and *NTNG2*, which encode neuron-related proteins. The *OR8D1* (2.47-fold change) and *OR10G9* (2.07-fold change) genes encode olfactory receptors 8D1 and 10G9, respectively, whereas *NTNG2* (2.11-fold change) encodes the netrin-G2 precursor. Moreover, 2 transporter-related genes of the *in vitro* M cells were remarkably upregulated. The *MICU2* (2.36-fold change) gene encodes calcium uptake protein 2, and *SLC28A1* (2.32-fold change) encodes a nucleoside transporter. The only significantly downregulated gene, *VCX2*, encodes variable charge X-linked protein 2 (−2.463-fold change, Table 1).

**Table 1:** Significantly upregulated or downregulated genes of *in vitro* M cells compared with Caco-2 cells.

Gene	Product	Description	Fold change
**Scaffold**			
** *HSCHR7_CTG4_4***	Hypothetical	Chr7:155062052–155061993 Between *HTR5A-AS1* (*HTR5A* antisense RNA 1) and *PAXIP1-AS1* (*PAXIP1* antisense RNA 1)	17.637
** *HSCHR9_CTG35***	Hypothetical	Chr9:109401669–109435160 Close to *PTPN3* (protein tyrosine phosphatase, nonreceptor type 3)	8.860
** *HSCHR6_CTG5***	Hypothetical		2.179
** *HSCHR15_CTG8***	Hypothetical		2.153
** *HSCHR1_CTG32_1***	Hypothetical		2.082
** *HSCHR10_CTG2***	Hypothetical	Chr10:3309069–3309128 Between *PITRM1* (pitrilysin metallopeptidase 1) and *LOC101927880* (lncRNA)	2.062
**Long noncoding RNA**			
** *LINC01333***	Hypothetical	Unknown	2.622
** *LINC00841***	Hypothetical	Unknown	2.542
** *LINC00689***	Hypothetical	Unknown	2.455
** *FLJ36000***	Hypothetical	Unknown	2.178
** *LOC100132099***	Hypothetical	Unknown	2.120
** *LOC101929450***	Hypothetical	Unknown	2.117
** * LOC284889***	Hypothetical	Unknown	2.038
**Membrane association**			
** *PITPNB***	Phosphatidylinositol transfer protein β	Catalyze the transfer of phosphatidylinositol and phosphatidylcholine	2.086
**Neuron-related protein**			
** *OR8D1***	Olfactory receptor, family 8, subfamily D, member 1	Mediate neurological system development	2.477
** *NTNG2***	Netrin-G2	Maintain glutamatergic neural circuitry	2.117
** *OR****10G****9***	Olfactory receptor, family 10, subfamily G, member 9	Mediate neurological system development	2.075
**Transporter**			
** *MICU2***	Mitochondrial calcium uptake 2	Calcium uptake protein	2.365
** *SLC****28A****1***	Solute carrier family 28	Nucleoside transporter	2.321
**Uncharacterized**			
** *VCX2***	Variable charge, X-linked 2	Belong to X-linked members	−2.463

### Microarray analysis identifies 25 significantly upregulated genes and 1 significantly downregulated gene of *S*. Typhimurium WT-infected *in vitro* M cells compared with Caco-2 cells

We investigated the impacts of *S*. Typhimurium infection on *in vitro* M cells and Caco-2 cells by comparing the transcriptomes of WT *S*. Typhimurium-infected cells with those of uninfected cells after analysis conducted using RNA microarrays. Twenty-six genes of Caco-2 cells were significantly regulated after WT *S*. Typhimurium infection, including 8 scaffold genes (2.646- to 2.059-fold change), 3 lncRNA genes (2.076- to −2.146-fold change), 2 membrane remodeling associated genes (2.169- to 2.379-fold change), 3 neuron-related genes (2.022- to 2.472- fold change), 9 inflammation-related genes (2.112- to 10.083-fold change), and one uncharacterized gene encoding a leucine-rich protein (S2 Table). The functions of the proteins encoded by all the identified scaffold and lncRNA genes were unknown. Regarding the 2 upregulated genes associated with membrane remodeling, *MYL4* encodes myosin light chain 4, which is a hexametric ATPase cellular motor protein, whereas *SCTR* encodes a secretin receptor that belongs to the G protein-coupled receptor family. Moreover, the 3 neuron-related genes were upregulated, including *ZFP36* encoding zinc finger protein 36, which is a myeloid cell tristetraprolin (TTP) mediating the regulation of myeloid cell differentiation, *GABRQ* encoding γ-aminobutyric acid (GABA) A receptor θ, which mediates neurotransmission, and *OPALIN* encoding oligodendrocytic myelin paranodal and inner-loop protein, which controls the development of the central nervous system. The 9 upregulated inflammatory genes are those encoding chemokines interleukin (IL)-8, which mediates the chemoattraction of neutrophils, and CXCL2, NFKBIA, NFKBIZ, and TNFFAIP3 involved in the activation of NF-κB signaling, CPED1, which mediates cell adhesion, and IER3, tumor necrosis factor (TNF), and CELF4, which mediate apoptosis.

We classified 13 significantly upregulated or downregulated genes of *S*. Typhimurium-infected *in vitro* M cells into 3 groups (S3 Table). The most highly upregulated scaffold gene *HSCHR7_CTG4_4* in the uninfected *in vitro* M cells compared with the Caco-2 cells (Table 1) was reversely downregulated (−10.109-fold change) after WT *S*. Typhimurium infection (S3 Table). Similar to the upregulation in Caco-2 cells after WT *S*. Typhimurium infection (S2 Table), the neuron-related gene *ZFP36* was significantly upregulated (2.266-fold change) in the WT *S*. Typhimurium-infected *in vitro* M cells (S3 Table). This observation indicated that *ZFP36* is involved in *S*. Typhimurium infection. The inflammation-related genes constitute the master group of strongly regulated genes in the WT *S*. Typhimurium-infected *in vitro* M cells, with *IL8* as the most highly expressed gene (14.929-fold change) and the genes encoding chemokine ligands and NF-κB activators. The *JUN* (3.010-fold change) and *KLF6* (2.006-fold change) genes were significantly upregulated only in the WT *S*. Typhimurium-infected *in vitro* M cells (S3 Table), but neither in the WT *S*. Typhimurium-infected Caco-2 cells nor in the *S*. Typhimurium Δ*speG*-infected *in vitro* M cells or Caco-2 cells (S2, S4 and S5 Tables), indicating that *JUN* and *KLF6* are inflammatory factors specific to the *S*. Typhimurium-infected M cells and their expression requires presence of bacterial *speG*.

### Microarray analysis identifies 22 significantly upregulated genes and 4 significantly downregulated genes of *S*. Typhimurium Δ*speG*-infected *in vitro* M cells compared with Caco-2 cells

To explore the effects of the *speG* gene on *S*. Typhimurium-infected *in vitro* M cells and Caco-2 cells, we compared the transcriptomes of the *S*. Typhimurium Δ*speG*-infected *in vitro* M cells and Caco-2 cells against those of the corresponding uninfected cells. First, we performed microarray analysis to compare the gene expression levels of *S*. Typhimurium Δ*speG*-infected Caco-2 cells against those of uninfected Caco-2 cells (S4 Table). The scaffold genes *HSCHR19_CTG3_1* and *HSCHR3_CTG2_1* were significantly upregulated (2.147- to 2.11-fold change) in the *S*. Typhimurium Δ*speG*-infected Caco-2 cells, and both of these genes were also significantly upregulated in the WT *S*. Typhimurium-infected Caco-2 cells (S2 Table). The *LINC00841* (2.334-fold change) gene was the only upregulated lncRNA gene in Δ*speG*-infected Caco-2 cells. The neuron-related gene *ZFP36* was upregulated not only in the WT *S*. Typhimurium-infected *in vitro* M cells (S3 Table) and Caco-2 cells (S2 Table) but also in the *S*. Typhimurium Δ*speG*-infected Caco-2 cells (2.423-fold change, S4 Table). The inflammatory genes also comprised the major group of considerably regulated genes in the *S*. Typhimurium Δ*speG*-infected Caco-2 cells, with *IL8* being the most significantly upregulated gene (23.865-fold change, S4 Table). This *IL8* expression level of the *S*. Typhimurium Δ*speG*-infected Caco-2 cells was higher than that of the WT *S*. Typhimurium-infected Caco-2 cells (10.083-fold change, S2 Table). Without *speG*, *IL6* (3.12–fold change) and *TNF* (3.069-fold change) were significantly upregulated in the Caco-2 cells after *S*. Typhimurium infection, whereas *CELF4* was not significantly upregulated (S2 and S4 Tables), suggesting that *speG* is involved in upregulation of *IL6* and *TNF* and downregulation of *CELF4* in the *S*. Typhimurium-infected Caco-2 cells.

Second, we compared the transcriptome of the *S*. Typhimurium Δ*speG*-infected *in vitro* M cells with that of the uninfected *in vitro* M cells (S5 Table). Similar to that (−10.109-fold change, S3 Table) in the WT *S*. Typhimurium-infected M cells, the scaffold gene *HSCHR7_CTG4_4* was significantly downregulated (−12.496-fold change, S5 Table) in the *S*. Typhimurium Δ*speG*-infected *in vitro* M cells. Regardless of cell types or the presence of *speG* in *S*. Typhimurium, the neuron-related gene *ZFP36* was upregulated in the *S*. Typhimurium Δ*speG*-infected *in vitro* M cells (2.009-fold change, S5 Table). These findings indicated that the upregulation of *ZFP36* was chiefly due to *S*. Typhimurium, not due to *speG* or the cell type. The *IL8* gene was the most significantly upregulated of the *S*. Typhimurium Δ*speG*-infected *in vitro* M cells (22.356-fold change, S5 Table). The potassium transporter gene *KCTD11* of the *in vitro* M cells was markedly upregulated after Δ*speG* infection (S5 Table), indicating that *speG* plays a role in suppressing the expression of this potassium transporter of M cells during *S*. Typhimurium infection.

### Microarray analysis is validated by qRT-PCR in the mRNA expression of *HSCHR7_CTG4_4*, *PITPNB*, *OR8D1*, *ZFP36*, *JUN*, *KCTD11*, and *IL6* of *in vitro* M cells and Caco-2 cells after *S*. Typhimurium WT and Δ*speG* infection

**Fig 3:**
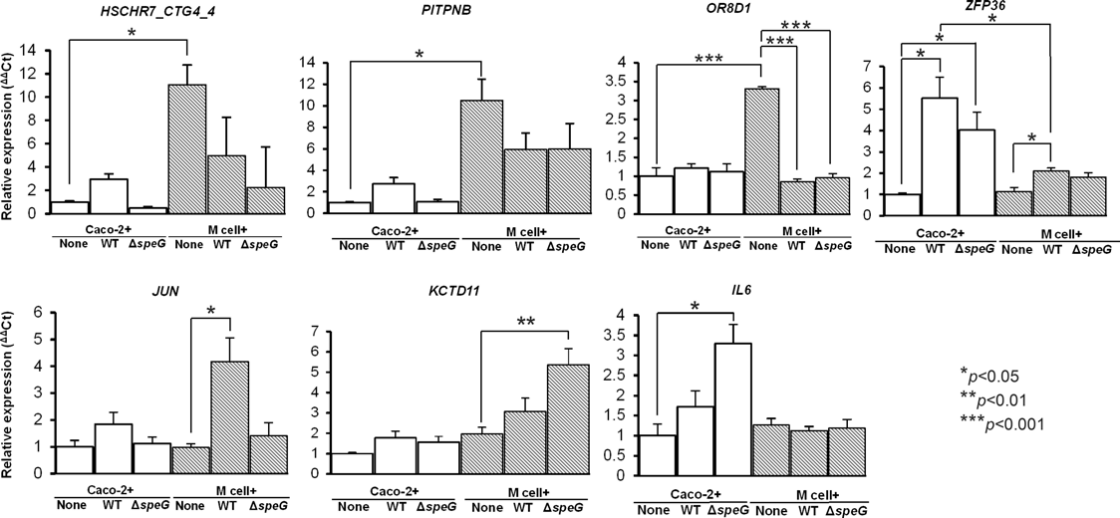
Quantitative real-time polymerase chain reaction (qRT-PCR) analysis of 7 selected genes, *HSCHR7_CTG4_4*, *PITPNB*, *OR8D1*, *ZFP36*, *JUN*, *KCTD11*, and *IL6* in the uninfected, wild-type (WT) *S*. Typhimurium- and Δ*speG*-infected Caco-2 cells and *in vitro* M cells. The 7 significantly regulated genes indentified using RNA microarrays were further validated through qRT-PCR with their specific primers. The mRNA expression levels of each group were normalized against the geometric means of 3 house keeping genes *GAPDH*, *RPLP* and *HPRT*. The expression levels of individual genes were calculated using the ^ΔΔ^Ct method, and expressed as the fold change compared with the geometric mean expression level of uninfected Caco-2 cells in triplicate. Data are represented as mean. ± SEM. *p* < 0.05 was considered to represent a significant difference.

To reconfirm the microarray data, we selected 7 significantly regulated genes for qRT-PCR to quantify their mRNA expression levels in the *in vitro* M cells and Caco-2 cells with and without infection of *S*. Typhimurium WT and Δ*speG*. The earlier microarray data showed that the scaffold gene *HSCHR7_CTG4_4* was significantly upregulated in uninfected *in vitro* M cells compared with uninfected Caco-2 cells (Table 1), and remarkably downregulated in the WT *S*. Typhimurium- and Δ*speG*-infected *in vitro* M cells (S3 Table). The qRT-PCR analysis results revealed reproducible data showing that *HSCHR7_CTG4_4* was highly expressed in the uninfected *in vitro* M cells (11.5-fold higher than in the uninfected Caco-2 cells); however, the mRNA expression levels were significantly inhibited in the *in vitro* M cells after WT *S*. Typhimurium and Δ*speG* infection (Fig 3). In addition, the microarray data indicated that both the *PITPNB* and *OR8D1* genes were significantly upregulated in uninfected *in vitro* M cells (Table 1), which was similarly shown by a 10- and 3.3-fold change in the upregulation of *PITPNB* and *OR8D1*, respectively, in qRT-PCR analysis (Fig 3). The expression levels of *OR8D1* were significantly downregulated in the *in vitro* M cells after *S*. Typhimurium WT and Δ*speG* infection compared with the uninfected cells according to qRT-PCR analysis (Fig 3); however, they were nonsignificantly downregulated (−1.197- and −1.339-fold change, respectively) according to the microarray data. The gene expression of *ZFP36* was significantly upregulated in the *in vitro* M cells and Caco-2 cells after both WT *S*. Typhimurium and Δ*speG* infection according to both qRT-PCR (Fig 3) and microarray analyses (S2 to S5 Tables). The expression levels of *ZFP36* in *S*. Typhimurium-infected M cells were significantly lower than those in *S*. Typhimurium-infected Caco-2 cells regardless of *speG* deletion (Fig 3), suggestive of milder *ZFP36*-related *speG*-independent inflammation in M cells than in Caco-2 cells. Moreover, the major inflammatory gene *JUN* was significantly upregulated only in the WT *S*. Typhimurium-infected *in vitro* M cells, but neither in the WT *S*. Typhimurium-infected Caco-2 cells nor in the *S*. Typhimurium Δ*speG*-infected Caco-2 cells or *in vitro* M cells (Fig 3), implying that *JUN* is a characteristic inflammatory factor of M cells after *S*. Typhimurium infection and requires contribution of *speG* to such inflammation. The potassium transporter gene *KCTD11* was significantly upregulated only in the *S*. Typhimurium Δ*speG*-infected *in vitro* M cells, and *IL6* was highly expressed in the *S*. Typhimurium Δ*speG*-infected Caco-2 cells (Fig 3). The significant upregulation of these 3 genes, as determined through qRT-PCR analysis, was consistent with the microarray analysis results.

## Discussion

We successfully established an *in vitro* M-cell culture model to investigate the differences in the expression of several previously unreported genes in *in vitro* M cells when compared with Caco-2 cells; we identified a significant expression of these genes for the first time in human *in vitro* M cells. Researchers using rat models have indicated that Slug, RANKL, and SpiB are involved in M-cell development [5, 7]. However, none of the genes related to these 3 markers were significantly upregulated in our human *in vitro* M-cell model, implying the possible presence of host specificity in M cells. The phosphoinositide 3-kinase (PI3K) signaling pathway may play a potential role in M cells. Our microarray data revealed that *HSCHR9_CTG35*, which is located near the genes *PTPN3* (Table 1), and *PITPNB* were significantly upregulated in our human *in vitro* M cells. The proteins encoded by *PTPN3* and *PITPNB* might be involved in PI3K regulation [23, 24]. Studies have indicated that PI3K and Wnt/β-catenin signaling pathways contribute to cell differentiation. For example, the development of lymphocytes is modulated by PI3K-related signals [25]. This evidence hints that the differentiation of human *in vitro* M cells is also controlled via the PI3K signaling pathway. Moreover, several neuron-related genes, including *NTNG2*, *OR8D1*, and *OR10G9*, were strongly upregulated after the transformation of Caco-2 cells into *in vitro* M cells in our model (Table 1). The *NTNG2* gene encodes the netrin-G2 protein, which is a potential mediator for mediating the development of the *Drosophila* olfactory system [26]. Moreover, the *OR8D1*- and *OR10G9*-encoded olfactory receptor proteins are potentially involved in olfactory system development. To date studies have not linked these genes with human M cells; nevertheless, *MICU2*, which encodes mitochondrial calcium uptake 2 protein, can regulate mitochondrial activity and cell survival [27], and *SLC28A1*, which encodes solute carrier family 28, a nucleoside transporter, were markedly upregulated in our *in vitro* M cells. The solute carrier family also potentially regulates mitochondrial activity [28]. Because mitochondrial activity is closely related to the differentiation of cell embryonal and stem cells [29], mitochondrial activity might be implicated in development of M cells. The only one significantly downregulated gene, *VCX2*, which encodes an uncharacterized X-linked member protein, might counteract the differentiation of human *in vitro* M cells. To date, X-linked members have not been characterized in M-cell development. The B cells from Peyer’s patches of X-linked immunodeficient mice distinctly interact with T cells [30]. Therefore, the X-linked members possibly regulate the microenvironment of gut immunity. These novel findings warrant further investigation for clarification.

Human M-cell makers have rarely been reported, except sialyl Lewis A antigen and galectin-9, which have been identified in M cells in human intestinal tissues [31, 32]. However, the functions of these 2 markers remain unclear. In addition, several M cell-specific proteins have been used as specific markers for identifying M cells in animal studies, with limited knowledge on their features, such as secretory granule neuroendocrine protein 1 (Sgen-1) [33], annexin A5 [34] and glycoprotein 2 (GP2) in mice [35], vimentin in rabbits [36], and cytokeratin 18 in cattle [37]. The GP2, expressed on the apical sides of murine M cells, has been well documented in previous studies. By contrast, GP2 remained undetected in a study using human *in vitro* M cells [38], as well as in our microarray data. Therefore, GP2 might be a poor marker for identifying human *in vitr*o M cells, or even genuine human M cells. Our results revealed that the scaffold sequence *HSCHR7_CTG4_4* exhibited the highest expression level in the transcriptome of the *in vitro* M cells (17.637-fold change, Table 1), which was confirmed by the similarly high mRNA expression level identified through qRT-PCR analysis (10.14-fold change, Fig 3). However, *HSCHR7_CTG4_4* was significantly downregulated after *S*. Typhimurium infection, regardless of the presence of *speG* (−10.109-fold change, S3 Table; −12.496-fold change, S5 Table); that is, *HSCHR7_CTG4_4* of human *in vitro* M cells can be *speG*-independently switched off by *S*. Typhimurium infection (green versus red bricks in Fig 2). In summary, *HSCHR7_CTG4_4* can be strongly expressed noninfectiously and suppressed postinfectiously in human *in vitro* M cells. It is worthy of investigation whether such a drastic change from upregulation to downregulation of *HSCHR7_CTG4_4* could transform more Caco-2 cells to M-like cells in our *in vitro* M-cell model after *S*. Typhimurium infection. The function of *HSCHR7_CTG4_4*, which encodes a hypothetical protein, is estimated to be similar to that of its neighboring genes *HTR5A-AS1* and *PAXIP1-AS1*. *HTR5A-AS1* is involved in serotonin receptor formation and *PAXIP1-AS1* is involved in the expression of PAX-interacting protein 1, which encodes a nuclear protein with 6 breast cancer carboxy-terminal (BRCT) domains for maintaining genome stability, chromatin condensation, and progression through mitosis. Another gene, *HSCHR9_CTG35*, also had a similar gene expression to that of *HSCHR7_CTG4_4* in human *in vitro* M cells (Table 1 and Fig 2), and this gene is located near *PTPN3*, which encodes a member of the protein tyrosine phosphatase family for the regulation of cellular processes and membrane-related functions. Although the phenotypes of the proteins encoded by these 2 genes remain hypothetical, the mentioned functions of their neighboring genes could possibly be involved in human M-cell development. Their encoded proteins can be potential candidate cell markers for identifying and isolating human M cells in the future. Moreover, *S*. Typhimurium infection can induce the transition of bovine and murine intestinal epithelial cells to M cells via activation of RANKL [6], but the expression of the RANKL genes were not significantly upregulated in the human Caco-2 cells and *in vitro* M cells after *S*. Typhimurium infection in our study. Therefore, the transformation of M cells might not be entirely controlled by the same regulatory networks in different species.

In the host, M cells provide a crucial route for bacterial internalization and transcytosis to the lamina propria. After such transcytosis across the intestinal epithelium, bacteria might encounter lymphocytes for antigen presentation to T cells and mononuclear phagocytes for the clearance of antigens [39]. Differing from the translocation of antigens across M cells into their basolateral space, intestinal epithelial cells secrete the IL-8 chemokine to attract neutrophils for migrating into the basolateral side of the infected intestinal epithelium and the subsequent clearance of bacteria [40]. IL-8 is involved in *Salmonella* internalization, and its transcription and secretion in the epithelium is activated via the NF-κB pathway [41]. Our data also revealed that *IL8* was highly upregulated in both *in vitro* M cells and Caco-2 cells after *S*. Typhimurium infection, with the simultaneous upregulation of the genes related to the NF-κB signaling pathway for the activation of inflammatory responses. Another study also reported that *IL8* transcription in *in vitro* M cells was upregulated after *Escherichia coli* and *Bacteroides fragilis* infections [42]; however, to date, no such report has been presented on salmonellosis. We speculated that IL-8 released from M cells might attract more gathering of immune cells beneath M cells to facilitate elimination of transcytosed bacteria. Moreover, *JUN*, which encodes the other major transcription factor AP-1, related to the activation of immunity, was highly expressed only in the *S*. Typhimurium-infected *in vitro* M cells according to our microarray data, implicating the significance of *JUN* in M cells after *S*. Typhimurium infection. Furthermore, a recent study using single-cell RNA-sequencing showed that the expression of type I interferon and toll-like receptor 4 related genes was upregulated within macrophages isolated from *S*. Typhimurium-infected mice [43]. However, the upregulated expression of these genes was not observed in our *in vitro* M cells and Caco-2 cells infected with *S*. Typhimurium. The contrasting results between phagocytic and non-phagocytic cells suggested the diversity of immune responses in different host cells after *Salmonella* infection.

Although *in vitro* M cells are derived from Caco-2 cells, our findings indicated that the immune responses of both cells can differ and involve *speG*. One study indicated that an SPI-1 factor of *Salmonella*, SipA, can induce CXC-chemokine expression in HeLa cells via the JUN pathway [44]. *Salmonella* can provoke membrane ruffling of host cells through its SPI- 1 factors during invasion into epithelial cells. The membrane ruffling results from cytoskeletal rearrangement, which is induced by G-coupled protein activation [45], and potentially regulated by ATPase [46]. Our microarray analysis results consistently showed that the ATPase-encoding gene *MYL4* and the G-coupled protein-encoding gene *SCTR* were significantly upregulated in Caco-2 cells after *S*. Typhimurium infection but not in M cells, indication that *MYL4* and *SCTR* are *speG*-dependent inflammatory factors specific to the *S*. Typhimurium-infected Caco-2 cells. In addition, the involvement of *speG* in upregulation of *IL6* and *TNF* and downregulation of *CELF4* in the *S*. Typhimurium-infected Caco-2 cells is also validated by our microarray analysis. By contrast, our data showed that *JUN* encoding transcription factor AP-1 and *KLF6* encoding Kruppel-like factor 6 were significantly upregulated in M cells rather than in Caco-2 cells after *S*. Typhimurium infection with presence of *speG*. In addition, potassium transporter-encoding *KCTD11* was remarkably downregulated in M cells after *S*. Typhimurium infection with absence of *speG*. Therefore, *speG* can modulate the expression of *JUN*, *KLF6*, and *KCTD11* in the *S*. Typhimurium-infected M cells.

The inflammation-related gene expression of *in vitro* M cells and Caco-2 cells after *S*. Typhimurium infection was not attenuated, but augmented by the deletion of *Salmonella speG*, which disabled intracellular *S*. Typhimurium from proliferating within host cells. Our preliminary study indicated the incapability of intracellular replication, but the capability of bacterial invasion of *S*. Typhimurium Δ*speG* in HeLa cells [18]. The expression of *speG* of *S*. Typhimurium was downregulated by the infection-relevant growth conditions such as late stationary phase, cold shock, pH shock, NaCl shock, anaerobic shock, peroxide shock, and nitric oxide shock that simulate environmental stressors during the infection of the mammalian host [14]. By contrast, the expression of *speG* was 1.3-fold expressed within macrophages [15], In addition, We found that the expression of *speG* was 1.6-fold increased in *S*. Typhimurium within the infected Caco-2 cells in comparison with those extracellular *S*. Typhimurium before invasion (S1 Fig). Therefore, we supposed that *speG* of *Salmonella* plays an influential role in immune responses after bacterial invasion into host cells, and we conducted this *in vitro* study utilizing two human intestinal epithelial cells. We obtained the transcriptomes of uninfected *in vitro* M cells and Caco-2 cells, as well as those of both cells infected with WT *S*. Typhimurium and Δ*speG* by using RNA microarrays and performing a pairwise comparison. Most of the inflammation-related genes of *S*. Typhimurium Δ*speG*-infected cells were more significantly upregulated compared with those of WT *S*. Typhimurium-infected cells, either *in vitro* M cells or Caco-2 cells. Evidently, *IL8* of Caco-2 cells was the most strongly up-regulated among all of the genes examined after WT *S*. Typhimurium infection (10.083-fold change, S2 Table); however, such upregulation increased more than 2-fold after *S*. Typhimurium Δ*speG* infection (23.865-fold change, S4 Table). Moreover, the *IL8* expression of *in vitro* M cells was upregulated 14.929 fold after WT *S*. Typhimurium WT infection (S3 Table); however, this upregulation was increased approximately 1.5 times after *S*. Typhimurium Δ*speG* infection (22.356-fold change, S5 Table). The gene expression of the other inflammatory factors such as chemokine ligand 2, NF-κB inhibitors, and tumor necrosis factor (TNF) also exhibited a similar trend, but with milder intensity.

Furthermore, the disruption of *speG* in *S*. Typhimurium significantly upregulated the *IL6* expression of Caco-2 cells (3.120-fold change, S4 Table), but not upregulated in *in vitro* M cells (1.189-fold change, Fig 2), implying that *speG* can suppress *IL6* upregulation in *S*. Typhimurium-infected Caco-2 cells. Our qRT-PCR data showed a reproducible result (Fig 3). IL-6 is an integral cytokine mediator of the acute phase response to injury and infection, and can stimulate immune responses such as the activation of T cells proliferation [47]. Whether the intestinal epithelium is an important source for IL-6 production has rarely been reported. Increased IL-6 production by macrophages, T lymphocytes, and intestinal epithelial cells is a hallmark for the pathogenesis of inflammatory bowel disease [48]. The expression of IL-6 can be enhanced at the protein or gene levels in Caco-2 cells postinfection with *Salmonella* [49]. In addition, IL-6 can impair tight junctions, thereby increasing the permeability of Caco-2 cells [50]. Our study was the first report in no involvement of *IL6* upregulation in the inflammation of the *S*. Typhimurium-infected *in vitro* M cells that is different from the *speG*-involved inflammation of the *S*. Typhimurium-infected Caco-2 cells (Fig 3). The expression of *IL6* was downregulated in uninfected Caco-2 cells, and *IL6* was neutrally expressed after *S*. Typhimurium SL1344 infection and significantly upregulated after *S*. Typhimurium Δ*speG* infection (Figs 2 and 3). In addition, the *in vitro* M cells in our study exhibited no significant upregulation of *IL6* in the *S*. Typhimurium Δ*speG*-infected M cells (Figs 2 and 3). This implied that *IL6* expression was suppressed in a fully differentiated Caco-2 cells and *in vitro* M cells; however, *S*. Typhimurium initiated its expression, and the loss of *speG* further provoked its expression significantly in Caco-2 cells (Fig 3), and no such a phenomenon in M cells; that is, *S*. Typhimurium *speG* suppressed *IL6* expression and its related inflammation only in Caco-2 cells but not M cells. Overall, *speG* is crucial for suppressing IL-6-involved inflammation of human intestinal epithelial cells after *S*. Typhimurium infection, which is not a characteristic inflammatory factor of human M cells. Disabling *speG* in *S*. Typhimurium can stimulate *IL6*-related responses within Caco-2 cells, which might partly explain why *S*. Typhimurium Δ*speG* lost its capability of intracellular proliferation in our study.

The *ZFP36* encoding zinc finger protein 36, namely TTP, was significantly upregulated in Caco-2 cells and *in vitro* M cells after *S*. Typhimurium infection, regardless of the presence of *speG*. TTP can regulate the function of brain-derived neurotrophic factor in the central nervous system, and iron homeostasis in fibroblasts [51]. In addition, a recent study indicated that TTP has an anti-inflammatory effect on the IL-10 production of murine macrophages [52]. IL-10 can be secreted from human colon epithelial cells, and impede pathogen clearance by inhibiting the NF-κB signaling pathway in human monocytes [53]. Therefore, we proposed that *ZFP36* upregulation can suppress the IL-10 secretion of host cells, not only of phagocytic cells but possibly of intestinal epithelial cells and M cells as well after *S*. Typhimurium infection, and such a phenotype of *ZFP6* is not be influenced by the *Salmonella speG* gene. Our study demonstrated that *ZPF36* was significantly upregulated in the *S*. Typhimurium-infected Caco-2 cells and *in vitro* M cells with a higher intensity in the former (Fig 3), suggesting a hypothesis that milder *ZPF36* expression in M cells than in Caco-2 cells contributes to the functional or morphological characteristics of M cells. The *ZFP36* gene might be an important host defense factor of human intestinal epithelial cells, including M cells, during bacterial infection, and requires further study for elucidation.

Certain neuron-related genes of human intestinal epithelial cells are significantly expressed during *Salmonella* infection. Unlike *ZFP36* which was persistently upregulated in both *in vitro* M cells and Caco-2 cells after WT S. Typhimurium and Δ*speG* infection, the other 2 neuron-related genes, *GABRQ* and *OPALIN*, were significantly upregulated in only *S*. Typhimurium-infected Caco-2 cells (2.028- and 2.022-fold change, respectively; S2 Table), but not in uninfected or infected M cells (S3 and S5 Tables). The deletion of *speG* in *S*. Typhimurium normalized the upregulation of *GABRQ* and *OPALIN* in Caco-2 cells (S4 Table), implying that *speG* modulates the expression of these 2 genes in Caco-2 cells after *S*. Typhimurium infection. *GABRQ* encodes GABA receptor A, which is involved in synaptic transmission within the neural system [54]. Because GABA plays a role in anti-inflammation and the inhibition of autoimmune inflammation in mice [55], *GABRO* upregulation in *S*. Typhimurium-infected Caco-2 cells implied that *GABRO* is possibly involved in host cell defense of intestinal epithelium during salmonellosis. In addition, *OPALIN* mediates development of the central nervous system and its encoding protein Opalin, also known as transmembrane protein 10, is a specific marker in oligodendrocytes, which can regulate microglial activity via the TNF-α and IL-1β regulatory network in mice and rats [56]. To date, no study has reported any link between *OPALIN* and infection or inflammation of the intestinal epithelium. Our novel findings of these 2 neuron-related genes and the contribution of *speG* to their upregulation warrant further investigation for clarification into their roles in gut immunity.

We identified 11 individual lncRNA sequences in our microarray analysis, implying that the upregulation of these sequences may play a role in the regulation of *in vitro* M-cell development (Table 1), host responses of Caco-2 cells after *S*. Typhimurium infection (S2 Table), and a potential effect of *speG* on *S*. Typhimurium-infected Caco-2 cells (S4 Table). The lncRNAs have been wildly identified in the human genome. Approximately 62% of the identified genes may be transcribed into noncoding RNA, and approximately 14 000 lncRNA genes have been annotated in humans [57]. However, numerous lncRNAs have not been characterized in their functions. Several lncRNAs are involved in the regulation of immune response, including *NEAT*, which can mediate *IL8* expression [58], and *THRIL*, which can regulate TNF-α expression [59]. Nevertheless, the functions of the 11 identified lncRNAs in our microarrays remain unknown.

In conclusion, we successfully utilized an *in vitro* M-cell model to discover a number of novel genetic loci with significantly regulated gene expression after comparing and statistically analyzing the transcriptomes of *S*. Typhimurium-infected and uninfected *in vitro* M cells and Caco-2 cells. Significantly expressed *HSCHR7_CTG4_4* of uninfected *in vitro* M cells can be *speG*-independently suppressed by *S*. Typhimurium infection that is a remarkable change in the regulation of gene expression in M cells after *S*. Typhimurium infection, which represents an important but unreported cell characteristic. The immune responses of *in vitro* M cells and Caco-2 cells can differ and reply on *speG* or not, with *speG*-dependent regulation of *KYL4*, *SCTR*, *IL6*, *TNF*, and *CELF4* of Caco-2 cells, and *JUN*, *KLF6*, and *KCTD11* in M cells, or *speG*-independent modulation of *ZFP36* in both cells. This study provides a global gene profiling of *in vitro* M cells before and after *S*. Typhimurium infection, which can be developed as a platform for examining the *S*. Typhimurium Δ*speG* mutant as a candidate oral vaccine vector and its relevant gut immune responses.

## Supporting Information

S1 Fig The mRNA expression levels of *speG* of *S*. Typhumurium SL1344 in mid-log culture and in Caco-2 cells after 6-h infection by qRT-PCR analysis after normalization against 16s mRNA expression. (TIF)

S1 Table Primers for qRT-PCR analysis. (DOC)

S2 Table Significantly upregulated or downregulated genes of *S*. Typhimurium SL1344-infected Caco-2 cells compared with uninfected Caco-2 cells. (DOC)

S3 Table Significantly upregulated or downregulated genes of *S*. Typhimurium Sl1344-infected *in vitro* M cells compared with uninfected *in vitro* M cells. (DOC)

S4 Table Significantly upregulated genes of *S*. Typhimurium Δ*speG*-infected Caco-2 cells compared with uninfected Caco-2 cells. (DOC)

S5 Table Significantly upregulated or downregulated genes of *S*. Typhimurium Δ*speG*-infected *in vitro* M cells compared with uninfected *in vitro* M cells. (DOC)

## Abbreviations

BCRC: Bioresource Collection and Research Center
BRCT: breast cancer carboxy-terminal
CFU: bacterial colony-forming unit
DMEM: Dulbecco’s modified Eagle’s medium
EMT: epithelial–mesenchymal transition
FAE: follicle-associated epithelium
FBS: fetal bovine serum
GP2: glycoprotein 2
IL: interleukin
LB: Luria–Bertani
lncRNA: long noncoding RNA
M: microfold or memembraous
MOI: multiplicity of infection
NF-κB: receptor activator of nuclear factor-κB
PI3K: phosphoinositide 3-kinase
qRT-PCR: quantitative real-time polymerase chain reaction
RANKL: receptor activator of nuclear factor-κB ligand
*S*. Typhimurium: *Salmonella enterica* serovar Typhimurium
SEM: scanning electron microscopy
Sgen-1: secretory granule neuroendocrine protein 1
SPI: *Salmonella* pathogenicity island
T3SS: type III secretion system
TEER: transepithelial electrical resistance
TEM: transmission electron microscopy
TNF: tumor necrosis factor
TTP: tristetraprolin
WT: wild-type

## References

[1] Yamamoto M, Pascual DW, Kiyono H. M cell-targeted mucosal vaccine strategies. Curr Top Microbiol Immunol. 2012;354:39–52. 10.1007/82_2011_134 21688209 PMC7393565

[2] Jang MH, Kweon MN, Iwatani K, Yamamoto M, Terahara K, Sasakawa C, et al Intestinal villous M cells: an antigen entry site in the mucosal epithelium. Proc Natl Acad Sci U S A. 2004;101(16):6110–5. 10.1073/pnas.0400969101 15071180 PMC395931

[3] van der Flier LG, Clevers H. Stem cells, self-renewal, and differentiation in the intestinal epithelium. Annu Rev Physiol. 2009;71:241–60. 10.1146/annurev.physiol.010908.163145 18808327

[4] Rajasekaran SA, Beyenbach KW, Rajasekaran AK. Interactions of tight junctions with membrane channels and transporters. Biochim Biophys Acta. 2008;1778(3):757–69. 10.1016/j.bbamem.2007.11.007 18086552

[5] Kanaya T, Hase K, Takahashi D, Fukuda S, Hoshino K, Sasaki I, et al The Ets transcription factor Spi-B is essential for the differentiation of intestinal microfold cells. Nat Immunol. 2012;13(8):729–36. 10.1038/ni.2352 22706340 PMC3704196

[6] Tahoun A, Mahajan S, Paxton E, Malterer G, Donaldson DS, Wang D, et al *Salmonella* transforms follicle-associated epithelial cells into M cells to promote intestinal invasion. Cell Host Microbe. 2012;12(5):645–56. 10.1016/j.chom.2012.10.009 23159054

[7] Bolos V, Peinado H, Perez-Moreno MA, Fraga MF, Esteller M, Cano A. The transcription factor Slug represses E-cadherin expression and induces epithelial to mesenchymal transitions: a comparison with Snail and E47 repressors. J Cell Sci. 2003;116(Pt 3):499–511. 12508111 10.1242/jcs.00224

[8] de Lau W, Kujala P, Schneeberger K, Middendorp S, Li VS, Barker N, et al Peyer's patch M cells derived from Lgr5(+) stem cells require SpiB and are induced by RankL in cultured "miniguts". Mol Cell Biol. 2012;32(18):3639–47. 10.1128/MCB.00434-12 22778137 PMC3430189

[9] Sato S, Kaneto S, Shibata N, Takahashi Y, Okura H, Yuki Y, et al Transcription factor Spi-B-dependent and -independent pathways for the development of Peyer's patch M cells. Mucosal Immunol. 2013;6(4):838–46. 10.1038/mi.2012.122 23212199

[10] Kerneis S, Bogdanova A, Kraehenbuhl JP, Pringault E. Conversion by Peyer's patch lymphocytes of human enterocytes into M cells that transport bacteria. Science. 1997;277(5328):949–52. 9252325 10.1126/science.277.5328.949

[11] Gullberg E, Leonard M, Karlsson J, Hopkins AM, Brayden D, Baird AW, et al Expression of specific markers and particle transport in a new human intestinal M-cell model. Biochem Biophys Res Commun. 2000;279(3):808–13. 10.1006/bbrc.2000.4038 11162433

[12] Jones BD, Ghori N, Falkow S. *Salmonella typhimurium* initiates murine infection by penetrating and destroying the specialized epithelial M cells of the Peyer's patches. J Exp Med. 1994;180(1):15–23. 8006579 10.1084/jem.180.1.15 PMC2191576

[13] Haraga A, Ohlson MB, Miller SI. *Salmonellae* interplay with host cells. Nat Rev Microbiol. 2008;6(1):53–66. 10.1038/nrmicro1788 18026123

[14] Kroger C, Colgan A, Srikumar S, Handler K, Sivasankaran SK, Hammarlof DL, et al An infection-relevant transcriptomic compendium for *Salmonella enterica* Serovar Typhimurium. Cell Host Microbe. 2013;14(6):683–95. 10.1016/j.chom.2013.11.010 24331466

[15] Srikumar S, Kroger C, Hebrard M, Colgan A, Owen SV, Sivasankaran SK, et al RNA-seq Brings New Insights to the Intra-Macrophage Transcriptome of *Salmonella* Typhimurium. PLoS Pathog. 2015;11(11):e1005262 10.1371/journal.ppat.1005262 26561851 PMC4643027

[16] Kroger C, Dillon SC, Cameron AD, Papenfort K, Sivasankaran SK, Hokamp K, et al The transcriptional landscape and small RNAs of *Salmonella enterica* serovar Typhimurium. Proc Natl Acad Sci U S A. 2012;109(20):E1277–86. 10.1073/pnas.1201061109 22538806 PMC3356629

[17] Barbagallo M, Di Martino ML, Marcocci L, Pietrangeli P, De Carolis E, Casalino M, et al A new piece of the *Shigella* Pathogenicity puzzle: spermidine accumulation by silencing of the *speG* gene [corrected]. PLoS One. 2011;6(11):e27226 10.1371/journal.pone.0027226 22102881 PMC3213128

[18] Jelsbak L, Thomsen LE, Wallrodt I, Jensen PR, Olsen JE. Polyamines are required for virulence in *Salmonella enterica* serovar Typhimurium. PLoS One. 2012;7(4):e36149 10.1371/journal.pone.0036149 22558361 PMC3340349

[19] Fang SB. Early interactions of non-typhoidal *Salmonella* with human epithelium. Doctoral thesis, UCL (University College London) 2011.

[20] Martinez-Argudo I, Jepson MA. *Salmonella* translocates across an *in vitro* M cell model independently of SPI-1 and SPI-2. Microbiology. 2008;154(Pt 12):3887–94. 10.1099/mic.0.2008/021162-0 19047755

[21] Gust B, Challis GL, Fowler K, Kieser T, Chater KF. PCR-targeted *Streptomyces* gene replacement identifies a protein domain needed for biosynthesis of the sesquiterpene soil odor geosmin. Proc Natl Acad Sci U S A. 2003;100(4):1541–6. 10.1073/pnas.0337542100 12563033 PMC149868

[22] Jones MA, Hulme SD, Barrow PA, Wigley P. The *Salmonella* pathogenicity island 1 and *Salmonella* pathogenicity island 2 type III secretion systems play a major role in pathogenesis of systemic disease and gastrointestinal tract colonization of *Salmonella enterica* serovar Typhimurium in the chicken. Avian Pathol. 2007;36(3):199–203. 10.1080/03079450701264118 17497331

[23] Cosker KE, Shadan S, van Diepen M, Morgan C, Li M, Allen-Baume V, et al Regulation of PI3K signalling by the phosphatidylinositol transfer protein PITPα during axonal extension in hippocampal neurons. J Cell Sci. 2008;121(Pt 6):796–803. 10.1242/jcs.019166 18285448

[24] Venable CL, Frevert EU, Kim YB, Fischer BM, Kamatkar S, Neel BG, et al Overexpression of protein-tyrosine phosphatase-1B in adipocytes inhibits insulin-stimulated phosphoinositide 3-kinase activity without altering glucose transport or Akt/Protein kinase B activation. J Biol Chem. 2000;275(24):18318–26. 10.1074/jbc.M908392199 10751417

[25] Okkenhaug K, Vanhaesebroeck B. PI3K in lymphocyte development, differentiation and activation. Nat Rev Immunol. 2003;3(4):317–30. 10.1038/nri1056 12669022

[26] Das A, Chiang A, Davla S, Priya R, Reichert H, Vijayraghavan K, et al Identification and analysis of a glutamatergic local interneuron lineage in the adult Drosophila olfactory system. Neural Syst Circuits. 2011;1(1):4 10.1186/2042-1001-1-4 22330097 PMC3257541

[27] Mallilankaraman K, Doonan P, Cardenas C, Chandramoorthy HC, Muller M, Miller R, et al MICU1 is an essential gatekeeper for MCU-mediated mitochondrial Ca(^2+^) uptake that regulates cell survival. Cell. 2012;151(3):630–44. 10.1016/j.cell.2012.10.011 23101630 PMC3486697

[28] Fiermonte G, Paradies E, Todisco S, Marobbio CM, Palmieri F. A novel member of solute carrier family 25 (SLC25A42) is a transporter of coenzyme A and adenosine 3',5'-diphosphate in human mitochondria. J Biol Chem. 2009;284(27):18152–9. 10.1074/jbc.M109.014118 19429682 PMC2709381

[29] Mandal S, Lindgren AG, Srivastava AS, Clark AT, Banerjee U. Mitochondrial function controls proliferation and early differentiation potential of embryonic stem cells. Stem Cells. 2011;29(3):486–95. 10.1002/stem.590 21425411 PMC4374603

[30] Eldridge JH, Beagley KW, McGhee JR. Immunoregulation in the Peyer's patch microenvironment. Cellular basis for the enhanced responses by the B cells of X-linked immunodeficient CBA/N mice. J Immunol. 1987;139(7):2255–62. 2958543

[31] Giannasca PJ, Giannasca KT, Leichtner AM, Neutra MR. Human intestinal M cells display the sialyl Lewis A antigen. Infect Immun. 1999;67(2):946–53. 9916113 10.1128/iai.67.2.946-953.1999 PMC96409

[32] Pielage JF, Cichon C, Greune L, Hirashima M, Kucharzik T, Schmidt MA. Reversible differentiation of Caco-2 cells reveals galectin-9 as a surface marker molecule for human follicle-associated epithelia and M cell-like cells. Int J Biochem Cell Biol. 2007;39(10):1886–901. 10.1016/j.biocel.2007.05.009 17596995

[33] Hase K, Ohshima S, Kawano K, Hashimoto N, Matsumoto K, Saito H, et al Distinct gene expression profiles characterize cellular phenotypes of follicle-associated epithelium and M cells. DNA Res. 2005;12(2):127–37. 10.1093/dnares/12.2.127 16303744

[34] Verbrugghe P, Waelput W, Dieriks B, Waeytens A, Vandesompele J, Cuvelier CA. Murine M cells express annexin V specifically. J Pathol. 2006;209(2):240–9. 10.1002/path.1970 16552796

[35] Terahara K, Yoshida M, Igarashi O, Nochi T, Pontes GS, Hase K, et al Comprehensive gene expression profiling of Peyer's patch M cells, villous M-like cells, and intestinal epithelial cells. J Immunol. 2008;180(12):7840–6. 18523247 10.4049/jimmunol.180.12.7840

[36] Jepson MA, Mason CM, Bennett MK, Simmons NL, Hirst BH. Co-expression of vimentin and cytokeratins in M cells of rabbit intestinal lymphoid follicle-associated epithelium. Histochem J. 1992;24(1):33–9. 1372597 10.1007/BF01043285

[37] Hondo T, Kanaya T, Takakura I, Watanabe H, Takahashi Y, Nagasawa Y, et al Cytokeratin 18 is a specific marker of bovine intestinal M cell. Am J Physiol Gastrointest Liver Physiol. 2011;300(3):G442–53. 10.1152/ajpgi.00345.2010 21193527

[38] Mabbott NA, Donaldson DS, Ohno H, Williams IR, Mahajan A. Microfold (M) cells: important immunosurveillance posts in the intestinal epithelium. Mucosal Immunol. 2013;6(4):666–77. 10.1038/mi.2013.30 23695511 PMC3686595

[39] Peterson LW, Artis D. Intestinal epithelial cells: regulators of barrier function and immune homeostasis. Nat Rev Immunol. 2014;14(3):141–53. 10.1038/nri3608 24566914

[40] Hammond ME, Lapointe GR, Feucht PH, Hilt S, Gallegos CA, Gordon CA, et al IL-8 induces neutrophil chemotaxis predominantly via type I IL-8 receptors. J Immunol. 1995;155(3):1428–33. 7636208

[41] Gewirtz AT, Rao AS, Simon PO Jr., Merlin D, Carnes D, Madara JL, et al *Salmonella typhimurium* induces epithelial IL-8 expression via Ca(^2+^)-mediated activation of the NF-κB pathway. J Clin Invest. 2000;105(1):79–92. 10.1172/JCI8066 10619864 PMC382586

[42] Lapthorne S, Macsharry J, Scully P, Nally K, Shanahan F. Differential intestinal M-cell gene expression response to gut commensals. Immunology. 2012;136(3):312–24. 10.1111/j.1365-2567.2012.03581.x 22385384 PMC3385031

[43] Avraham R, Haseley N, Brown D, Penaranda C, Jijon HB, Trombetta JJ, et al Pathogen Cell-to-Cell Variability Drives Heterogeneity in Host Immune Responses. Cell. 2015;162(6):1309–21. 10.1016/j.cell.2015.08.027 26343579 PMC4578813

[44] Figueiredo JF, Lawhon SD, Gokulan K, Khare S, Raffatellu M, Tsolis RM, et al *Salmonella enterica* Typhimurium SipA induces CXC-chemokine expression through p38MAPK and JUN pathways. Microbes Infect. 2009;11(2):302–10. 10.1016/j.micinf.2008.12.005 19114119

[45] Ma AD, Metjian A, Bagrodia S, Taylor S, Abrams CS. Cytoskeletal reorganization by G protein-coupled receptors is dependent on phosphoinositide 3-kinase γ, a Rac guanosine exchange factor, and Rac. Mol Cell Biol. 1998;18(8):4744–51. 9671484 10.1128/mcb.18.8.4744 PMC109060

[46] Ma B, Qian D, Nan Q, Tan C, An L, Xiang Y. Arabidopsis vacuolar H^+^-ATPase (V-ATPase) B subunits are involved in actin cytoskeleton remodeling via binding to, bundling, and stabilizing F-actin. J Biol Chem. 2012;287(23):19008–17. 10.1074/jbc.M111.281873 22371505 PMC3365934

[47] Takeda K, Kaisho T, Yoshida N, Takeda J, Kishimoto T, Akira S. Stat3 activation is responsible for IL-6-dependent T cell proliferation through preventing apoptosis: generation and characterization of T cell-specific Stat3-deficient mice. J Immunol. 1998;161(9):4652–60. 9794394

[48] Mitsuyama K, Sata M, Rose-John S. Interleukin-6 trans-signaling in inflammatory bowel disease. Cytokine Growth Factor Rev. 2006;17(6):451–61. 10.1016/j.cytogfr.2006.09.003 17045835

[49] Huang FC. Upregulation of *Salmonella*-induced IL-6 production in Caco-2 cells by PJ-34, PARP-1 inhibitor: involvement of PI3K, p38 MAPK, ERK, JNK, and NF-κB. Mediators Inflamm. 2009;2009:103890 10.1155/2009/103890 20204057 PMC2828125

[50] Suzuki T, Yoshinaga N, Tanabe S. Interleukin-6 (IL-6) regulates claudin-2 expression and tight junction permeability in intestinal epithelium. J Biol Chem. 2011;286(36):31263–71. 10.1074/jbc.M111.238147 21771795 PMC3173073

[51] Bayeva M, Khechaduri A, Puig S, Chang HC, Patial S, Blackshear PJ, et al mTOR regulates cellular iron homeostasis through tristetraprolin. Cell Metab. 2012;16(5):645–57. 10.1016/j.cmet.2012.10.001 23102618 PMC3594686

[52] Schaljo B, Kratochvill F, Gratz N, Sadzak I, Sauer I, Hammer M, et al Tristetraprolin is required for full anti-inflammatory response of murine macrophages to IL-10. J Immunol. 2009;183(2):1197–206. 10.4049/jimmunol.0803883 19542371 PMC2755621

[53] Couper KN, Blount DG, Riley EM. IL-10: the master regulator of immunity to infection. J Immunol. 2008;180(9):5771–7. 18424693 10.4049/jimmunol.180.9.5771

[54] Sigel E, Steinmann ME. Structure, function, and modulation of GABA(A) receptors. J Biol Chem. 2012;287(48):40224–31. 10.1074/jbc.R112.386664 23038269 PMC3504738

[55] Bhat R, Axtell R, Mitra A, Miranda M, Lock C, Tsien RW, et al Inhibitory role for GABA in autoimmune inflammation. Proc Natl Acad Sci U S A. 2010;107(6):2580–5. 10.1073/pnas.0915139107 20133656 PMC2823917

[56] Peferoen L, Kipp M, van der Valk P, van Noort JM, Amor S. Oligodendrocyte-microglia cross-talk in the central nervous system. Immunology. 2014;141(3):302–13. 10.1111/imm.12163 23981039 PMC3930369

[57] Derrien T, Johnson R, Bussotti G, Tanzer A, Djebali S, Tilgner H, et al The GENCODE v7 catalog of human long noncoding RNAs: analysis of their gene structure, evolution, and expression. Genome Res. 2012;22(9):1775–89. 10.1101/gr.132159.111 22955988 PMC3431493

[58] Imamura K, Imamachi N, Akizuki G, Kumakura M, Kawaguchi A, Nagata K, et al Long noncoding RNA NEAT1-dependent SFPQ relocation from promoter region to paraspeckle mediates IL8 expression upon immune stimuli. Mol Cell. 2014;53(3):393–406. 10.1016/j.molcel.2014.01.009 24507715

[59] Li Z, Chao TC, Chang KY, Lin N, Patil VS, Shimizu C, et al The long noncoding RNA THRIL regulates TNFα expression through its interaction with hnRNPL. Proc Natl Acad Sci U S A. 2014;111(3):1002–7. 10.1073/pnas.1313768111 24371310 PMC3903238

