# Supplementary figures and images for: Impacts of Salmonella enterica Serovar Typhimurium and Its speG Gene on the Transcriptomes of In Vitro M Cells and Caco-2 Cells
Source: PLoS One. 2016 Apr 11;11(4):e0153444. doi: 10.1371/journal.pone.0153444 (PMC4827826; doi:10.1371/journal.pone.0153444)

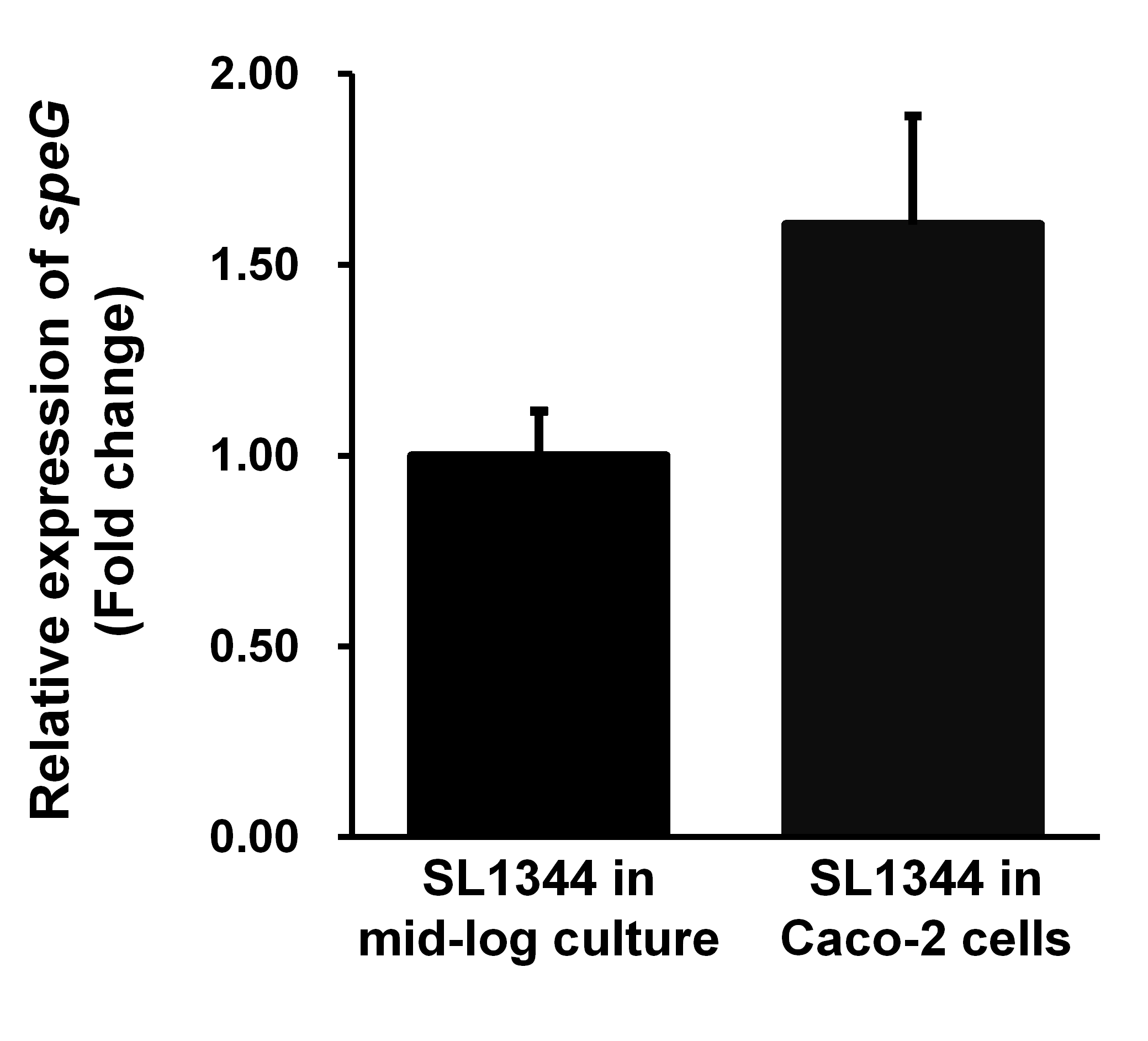

Supplement: S1 Fig — (TIF) [file pone.0153444.s001.tif]
