# Supplementary material for: Impacts of Salmonella enterica Serovar Typhimurium and Its speG Gene on the Transcriptomes of In Vitro M Cells and Caco-2 Cells
Source: PLoS One. 2016 Apr 11;11(4):e0153444. doi: 10.1371/journal.pone.0153444 (PMC4827826; doi:10.1371/journal.pone.0153444)
Supplement: S1 Table — (DOC) [file pone.0153444.s002.doc]

**S1 Table. Primers for qRT-PCR analysis**

| **Primer name**  **(F: forward, R: reverse)** | **Sequence (5′→3′)** | **Annealing temperature (°C)** | **Amplicon size (bp)** |
| --- | --- | --- | --- |
| ***CTG4_4*-F** | CTGTTTCTTTATCCACTCGTTGA | 55 | 113 |
| ***CTG4_4*-R** | TTTTAGATTTGGGAGCACTCTCT | 55 | 113 |
| ***PITPNB*-F** | TCCATCGCCAGCTTTTTT | 55 | 132 |
| ***PITPNB*-R** | CGCTTGTTCCCCTCACTT | 55 | 132 |
| ***OR8D1*-F** | CAGCCCTCTACTTCACACCC | 55 | 186 |
| ***OR8D1*-R** | TAACCCTCAGCCACCACAAA | 55 | 186 |
| ***ZFP36*-F** | GCTATGTCGGACCTTCTCA | 55 | 193 |
| ***ZFP36*-R** | GTCTTCGCTAGGGTTGTGG | 55 | 193 |
| ***JUN*-F** | TGCCTCCAAGTGCCGAAA | 55 | 128 |
| ***JUN*-R** | GCTGTGCCACCTGTTCCC | 55 | 128 |
| ***KCTD11*-F** | GCTCCACGCAGATGTAGAT | 55 | 125 |
| ***KCTD11*-R** | AGTCGGTGCAGAAAAGGTT | 55 | 125 |
| ***IL6*-F** | CATCACTGGTCTTTTGGAG | 55 | 159 |
| ***IL6*-R** | TCAGGGGTGGTTATTGCAT | 55 | 159 |
| ***GAPDH*-F** | AGGTCGGAGTCAACGGATTT | 55 | 220 |
| ***GAPDH*-R** | TGGAAGATGGTGATGGGATTT | 55 | 220 |
| ***RPLP0*-F** | GCAATGTTGCCAGTGTCTG | 55 | 140 |
| ***RPLP0*-R** | GCCTTGACCTTTTCAGCAA | 55 | 140 |
| ***HPRT1*-F** | TGACACTGGCAAAACAATGCA | 55 | 94 |
| ***HPRT1*-R** | GGTCCTTTTCACCAGCAAGCT | 55 | 94 |
| ***speG*-F** | ATCTCTACGACAAACACATCCA | 55 | 300 |
| ***speG*-R** | GCTCGCCTTCTACTCTAAACCC | 55 | 300 |
| ***16s*-F** | TTCCTCCAGATCTCTACGCA | 55 | 552 |
| ***16s*-R** | GTGGCTAATACCGCATAACG | 55 | 552 |
