# Supplementary material for: Impacts of Salmonella enterica Serovar Typhimurium and Its speG Gene on the Transcriptomes of In Vitro M Cells and Caco-2 Cells
Source: PLoS One. 2016 Apr 11;11(4):e0153444. doi: 10.1371/journal.pone.0153444 (PMC4827826; doi:10.1371/journal.pone.0153444)
Supplement: S2 Table — (DOC) [file pone.0153444.s003.doc]

**S2 Table. Significantly upregulated or downregulated genes of *S*. Typhimurium SL1344-infected Caco-2 cells compared with uninfected Caco-2 cells**

| **Gene** | **Product** | **Description** | **Fold change** |
| --- | --- | --- | --- |
| **Scaffold** |  |  |  |
| ***HSCHR10_CTG5*** | Hypothetical | Unknown | 2.646 |
| ***HSCHR3_CTG2_1*** | Hypothetical | Unknown | 2.280 |
| ***HSCHR16_CTG1*** | Hypothetical | Unknown | 2.222 |
| ***HSCHR15_CTG8*** | Hypothetical | Unknown | 2.164 |
| ***HSCHR17_CTG2*** | Hypothetical | Unknown | 2.160 |
| ***HSCHR19_CTG3_1*** | Hypothetical | Unknown | 2.130 |
| ***HSCHR11_CTG3_1*** | Hypothetical | Unknown | 2.065 |
| ***HSCHR9_CTG34*** | Hypothetical | Unknown | 2.059 |
| **Long noncoding RNA** |  |  |  |
| ***LOC101927289*** | Hypothetical | Unknown | 2.076 |
| ***RBM12B-AS1*** | Hypothetical | Unknown | 2.033 |
| ***LOC100506406*** | Hypothetical | Unknown | −2.146 |
| **Membrane association** |  |  |  |
| ***MYL4*** | Myosin, light chain 4 | ATPase cellular motor protein | 2.379 |
| ***SCTR*** | Secretin receptor | Belong to G protein-coupled receptor | 2.169 |
| **Neuron-related protein** |  |  |  |
| ***ZFP36*** | Zinc finger protein 36 | Mediate regulation of myeloid cell differentiation | 2.472 |
| ***GABRQ*** | γ-aminobutyric acid (GABA) A receptor, θ | Mediate neurotransmission | 2.028 |
| ***OPALIN*** | Oligodendrocytic myelin paranodal and inner-loop protein | Mediate development of the central nervous system | 2.022 |
| **Inflammation** |  |  |  |
| ***IL8*** | Interleukin 8 | Inflammatory factor | 10.083 |
| ***CXCL2*** | Chemokine (C-X-C motif) ligand 2 | Inflammatory factor | 8.307 |
| ***NFKBIA*** | NF-κB inhibitor α | Mediate activation of NF-κB | 3.972 |
| ***NFKBIZ*** | NF-κB inhibitor ζ | Mediate activation of NF-κB | 3.861 |
| ***TNFAIP3*** | Tumor necrosis factor α induced protein 3 | Mediate activation of NF-κB | 3.592 |
| ***CPED1*** | Cadherin-like and PC-esterase domain 1 | Mediate cell adhesion | 2.857 |
| ***IER3*** | Immediate early response 3 | Mediate activation of apoptosis | 2.736 |
| ***TNF*** | Tumor necrosis factor | Inflammatory and apoptotic factor | 2.706 |
| ***CELF4*** | CUGBP, Elav-like family member 4 | Mediate apoptosis | 2.112 |
| **Uncharacterized** |  |  |  |
| ***LRTOMT*** | Leucine-rich transmembrane and O-methyltransferase domain containing | Leucine-rich protein | 2.029 |
