# Supplementary material for: Impacts of Salmonella enterica Serovar Typhimurium and Its speG Gene on the Transcriptomes of In Vitro M Cells and Caco-2 Cells
Source: PLoS One. 2016 Apr 11;11(4):e0153444. doi: 10.1371/journal.pone.0153444 (PMC4827826; doi:10.1371/journal.pone.0153444)
Supplement: S3 Table — (DOC) [file pone.0153444.s004.doc]

**S3 Table. Significantly upregulated or downregulated genes of *S*. Typhimurium Sl1344-infected *in vitro* M cells compared with uninfected *in vitro* M cells**

| **Gene** | **Product** | **Description** | **Fold change** |
| --- | --- | --- | --- |
| **Scaffold** |  |  |  |
| ***HSCHR1_CTG3*** | Hypothetical | Unknown | −5.299 |
| ***HSCHR7_CTG4_4*** | Hypothetical | Chr7:155062052-155061993 Between *HTR5A-AS1* (*HTR5A* antisense RNA 1), *PAXIP1-AS1* (*PAXIP1* antisense RNA 1) | −10.109 |
| **Neuron-related protein** |  |  |  |
| ***ZFP36*** | Zinc finger protein 36 | Mediate regulation of myeloid cell differentiation | 2.266 |
| **Inflammation** |  |  |  |
| ***IL8*** | Interleukin 8 | Inflammatory factor | 14.929 |
| ***CXCL2*** | Chemokine (C-X-C motif) ligand 2 | Inflammatory factor | 10.341 |
| ***CXCL2*** | Chemokine (C-X-C motif) ligand 2 | Inflammatory factor | 8.878 |
| ***CXCL3*** | Chemokine (C-X-C motif) ligand 3 | Inflammatory factor | 6.653 |
| ***CXCL1*** | Chemokine (C-X-C motif) ligand 1 | Inflammatory factor | 6.330 |
| ***CXCL1*** | Chemokine (C-X-C motif) ligand 1 | Inflammatory factor | 5.846 |
| ***NFKBIZ*** | NF-κB inhibitor ζ | Mediate activation of NF-κB | 4.437 |
| ***NFKBIA*** | NF-κB inhibitor α | Mediate activation of NF-κB | 3.477 |
| ***JUN*** | Transcription factor AP-1 | Mediate expression of inflammatory factor | 3.010 |
| ***KLF6*** | Kruppel-like factor 6 | Inflammatory factor | 2.006 |
