# Supplementary material for: Impacts of Salmonella enterica Serovar Typhimurium and Its speG Gene on the Transcriptomes of In Vitro M Cells and Caco-2 Cells
Source: PLoS One. 2016 Apr 11;11(4):e0153444. doi: 10.1371/journal.pone.0153444 (PMC4827826; doi:10.1371/journal.pone.0153444)
Supplement: S4 Table — (DOC) [file pone.0153444.s005.doc]

**S4 Table. Significantly upregulated genes of *S.* Typhimurium Δ*speG*-infected Caco-2 cells compared with uninfected Caco-2 cells**

| **Gene** | **Product** | **Description** | **Fold change** |
| --- | --- | --- | --- |
| **Scaffold** |  |  |  |
| ***HSCHR19_CTG3_1*** | Hypothetical | Unknown | 2.147 |
| ***HSCHR3_CTG2_1*** | Hypothetical | Unknown | 2.110 |
| **Long noncoding RNA** |  |  |  |
| ***LINC00841*** | Hypothetical | Unknown | 2.334 |
| **Neuron-related protein** |  |  |  |
| ***ZFP36*** | Zinc finger protein 36 | Mediate regulation of myeloid cell differentiation | 2.423 |
| **Inflammation** |  |  |  |
| ***IL8*** | Interleukin 8 | Inflammatory factor | 23.865 |
| ***CXCL2*** | Chemokine (C-X-C motif) ligand 2 | Inflammatory factor | 13.987 |
| ***CXCL2*** | Chemokine (C-X-C motif) ligand 2 | Inflammatory factor | 12.547 |
| ***NFKBIZ*** | NF-κB inhibitor ζ | Mediate activation of NF-κB | 4.956 |
| ***NFKBIA*** | NF-κB inhibitor α | Mediate activation of NF-κB | 4.773 |
| ***TNFAIP3*** | Tumor necrosis factor α induced protein 3 | Mediate activation of NF-κB | 3.974 |
| ***IER3*** | Immediate early response 3 | Mediate activation of apoptosis | 3.293 |
| ***IL6*** | Interleukin 6 | Inflammatory factor | 3.120 |
| ***IER3*** | Immediate early response 3 | Mediate activation of apoptosis | 3.112 |
| ***TNF*** | Tumor necrosis factor | Inflammatory and apoptotic factor | 3.069 |
