# Supplementary material for: Impacts of Salmonella enterica Serovar Typhimurium and Its speG Gene on the Transcriptomes of In Vitro M Cells and Caco-2 Cells
Source: PLoS One. 2016 Apr 11;11(4):e0153444. doi: 10.1371/journal.pone.0153444 (PMC4827826; doi:10.1371/journal.pone.0153444)
Supplement: S5 Table — (DOC) [file pone.0153444.s006.doc]

**S5 Table. Significantly upregulated or downregulated genes of *S.* Typhimurium Δ*speG*-infected *in vitro* M cells compared with uninfected *in vitro* M cells**

| **Gene** | **Product** | **Description** | **Fold change** |
| --- | --- | --- | --- |
| **Scaffold** |  |  |  |
| ***HSCHR10_CTG2*** | Hypothetical | Chr10:3309069-3309128 Between *PITRM1* (pitrilysin metallopeptidase 1) and *LOC101927880* (LncRNA) | 4.327 |
| ***HSCHR6_CTG5*** | Hypothetical | Unknown | 2.233 |
| ***HSCHR3_CTG2_1*** | Hypothetical | Unknown | 2.172 |
| ***HSCHR12_CTG2_1*** | Hypothetical | Unknown | 2.021 |
| ***HSCHR15_CTG8*** | Hypothetical | Unknown | 2.017 |
| ***HSCHR22_CTG3_2*** | Hypothetical | Unknown | 2.004 |
| ***HSCHR4_CTG12*** | Hypothetical | Unknown | −2.359 |
| ***HSCHR1_CTG3*** | Hypothetical | Unknown | −5.145 |
| ***HSCHR9_CTG35*** | Hypothetical | Chr9:109401669-10943516 Close to PTPN3 (protein tyrosine phosphatase | −6.807 |
| ***HSCHR7_CTG4_4*** | Hypothetical | Chr7:155062052-15506199 Between *HTR5A-AS1* (*HTR5A* antisense RNA 1) and *PAXIP1-AS1* (*PAXIP1* antisense RNA 1) | −12.496 |
| **Neuron-related protein** |  |  |  |
| ***ZFP36*** | Zinc finger protein 36 | Mediate regulation of myeloid cell differentiation | 2.009 |
| **Inflammation** |  |  |  |
| ***IL8*** | Interleukin 8 | Inflammatory factor | 22.356 |
| ***CXCL2*** | Chemokine (C-X-C motif) ligand 2 | Inflammatory factor | 12.674 |
| ***CXCL2*** | Chemokine (C-X-C motif) ligand 2 | Inflammatory factor | 10.358 |
| ***CXCL1*** | Chemokine (C-X-C motif) ligand 1 | inflammatory factor | 8.629 |
| ***CXCL1*** | Chemokine (C-X-C motif) ligand 1 | Inflammatory factor | 7.077 |
| ***CXCL3*** | Chemokine (C-X-C motif) ligand 3 | Inflammatory factor | 6.513 |
| ***NFKBIZ*** | NF-κB inhibitor ζ | Mediate activation of NF-κB | 4.143 |
| ***NFKBIA*** | NF-κB inhibitor α | Mediate activation of NF-κB | 4.000 |
| ***TNFAIP3*** | Tumor necrosis factor α induced protein 3 | Mediate activation of NF-κB | 2.827 |
| ***IER3*** | Immediate early response 3 | Mediate activation of apoptosis | 2.651 |
| ***IER3*** | Immediate early response 3 | Mediate activation of apoptosis | 2.620 |
| **Transporter** |  |  |  |
| ***KCTD11*** | Potassium channel tetramerization domain containing 11 | Mediate potassium transportation | 2.022 |
| **Uncharacterized** |  |  |  |
| ***C2orf53*** | Chromosome 2 open reading frame 53 (hypothetical) | Unknown | 2.867 |
| ***FAM129C*** | Family with sequence similarity 129, member C | Niban-like protein 2 | 2.272 |
| ***C10orf53*** | Chromosome 10 open reading frame 53 (hypothetical) | Unknown | 2.033 |
